# Supplementary material for: Direct conversion of lignin to functionalized diaryl ethers via oxidative cross-coupling
Source: Nat Commun. 2023 May 22;14:2830. doi: 10.1038/s41467-023-38534-1 (PMC10203214; doi:10.1038/s41467-023-38534-1)
Supplement: Supplementary file 1 — Supplementary Information [file 41467_2023_38534_MOESM1_ESM.pdf]

Supplementary Information

Direct Conversion of Lignin to Functionalized Diaryl Ethers via Oxidative Cross-coupling

Mingyang Liu and Paul J. Dyson*

Institute of Chemical Sciences and Engineering, Ecole Polytechnique Fédérale de Lausanne (EPFL), 1015 Lausanne, Switzerland.

Contents

Supplementary Materials and methods 2
Supplementary table and figures 5
Supplementary NMR spectra 25
Supplementary References 47

Supplementary Materials and methods

Materials

Commercially available Cu salt catalysts were purchased from Alfa Aesar and co-catalysts (ligands) were purchased from Strem Chemicals. Bases, boronic acids, and other reagents were obtained from Sigma-Aldrich. Solvents were purchased from Acros Organics. Compressed air and O_2 was purchased from Carbagas. β–O–4 lignin model compounds were synthesized according to literature methods.[1] The extraction of organoslov lignin is based on an adapted literature method described below.[1]

Organoslov lignin extraction

Wood sawdust (< 0.5 mm) was dried at 80 ºC for 24 h prior to lignin extraction. Wood sawdust (50 g), dioxane (360 mL), and HCl aqueous solution (40 mL, 2 mol/L) were added to a flask and protected under N_2. The mixture was heated to reflux for 1 h (~110 ºC). The mixture was filtered, washed with dioxane and concentrated to afford a brown liquid-gel (~100 mL). The liquid-gel was dissolved in a mixture of acetone and H_2O (v/v = 9:1, 250 mL). Crude lignin was obtained by the precipitation of the solution in H_2O (2 L) and dried using lyophilizer. Crude lignin was dissolved in the mixture of acetone and methanol (v/v = 9:1, 200 mL, if not fully dissolved, further methanol may be added). The lignin was regenerated by precipitation in diethyl ether (2 L), filtered and dried under vacuum.

Synthesis of protected phenols

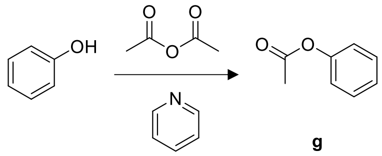

g

Acetylated phenol **g**:[2] Phenol (100 mmol), acetic anhydride (200 mmol, 2.0 equiv.) and pyridine (60 mmol, 0.6 equiv.) were mixed and stirred for 2 h at 25 ºC. After reaction, the mixture was diluted with toluene (100 mL) and washed with H_2O (3 × 100 mL). The organic phase was washed with brine, dried with anhydrous Mg_2SO_4, and finally concentrated in vacuo to give **g** as a colorless oil.

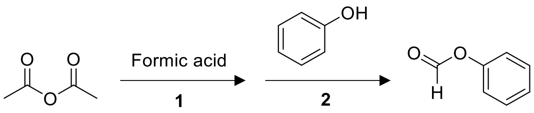

h

Formylated phenol **h**:[3] (1) Formic acid (500 mmol, 5.0 equiv.) and acetic anhydride (400 mmol, 4.0 equiv.) were stirred at 60 ºC for 1 h. (2) Phenol (100 mmol) and NaOAc (8.2 g, 100 mmol, 1.0 equiv.) were added into the solution at room temperature and stirred for a further 3.5 h. The product was purified as described above.

Typical reaction to prepare ether monomers from lignin

Beech lignin (40 mg, content of β–O–4 linkages is 0.245 mmol/g lignin), 4-chlorophenylboronic acid (1.5 equiv.), $Cu(OTf)_2$ (30 mol%), bathophenanthroline (L1, 30 mol%), K_2CO_3 (4 equiv.), biphenyl (internal standard, 2 equiv.), and DMSO (2 mL) were added into autoclave. The reactor was heated to 140 ºC for 6 h. After cooling to room temperature, the crude product was methylated by addition of K_2CO_3 (3 equiv.), MeI (10 equiv.) and stirring for 12 h. After reaction, the mixture was added ethyl acetate (EA, 3 mL) and a saturated NH_4Cl (5 mL) aqueous solution was added to extract the organic products. The aqueous solution was extracted into EA (3×3 mL). For qualitative and quantitative analysis of the products, the combined organic phase was filtered over silica gel to remove the inorganic salts and ligand. Further purification was achieved using column chromatography.

Analytical Methods

Qualitative and quantitative analysis of the crude products was performed by gas chromatography (GC) (Agilent 7890B, equipped with mass detector (Agilent 7000C) equipped with a hydrogen flame-ionization detector (FID) and HP-5 nonpolar column. The GC yield was determined based on internal standard curves and areas of integrated peak area.

^1H and ^{13}C NMR spectra were recorded on a Bruker Avance III HD 400 instrument equipped with a 5 mm BBFO probe. DMSO-d_6 or $CDCl_3$ were used as solvent. Short-range ^{13}C-^1H correlation NMR spectra (HSQC) were recorded on a 500 MHz spectrometer (Bruker AVANCENEO-500). HSQC spectra of original reaction mixture were recorded in DMSO-d_6, used as the reaction solvent. The reaction mixture was dried with 4A molecular sieve and centrifuged to remove inorganic salts.

Quantitative methods

Determination of the concentration of β–O–4 linkages by quantitative ^{13}C NMR spectroscopy would be hindered by the mixed signals of other alkyl linkages including the β–β and α–O–4 linkages.[4-5] Thus, we employed HSQC spectra with trioxane as an internal standard to determine the concentration of the β–O–4 linkages.[6] Mixtures of β–O–4 model compound and trioxane were tested to obtain a calibration curve and a correction factor of each functional group in lignin. Details are summaried in Supplementary Figs. 3-6.

Correction factor of α-H of β–O–4 linkages is:

$$f_{\beta\text{-}O\text{-}4} = 2.9613$$

The molar concentration (mmol/g) of β–O–4 linkages is:

$$M_{\beta\text{-}O\text{-}4} = \frac{6S_{\beta\text{-}O\text{-}4}}{S_{trioxane} f_{\beta\text{-}O\text{-}4}} \times \frac{m_{trioxane}}{M_w(trioxane)} \times \frac{1000}{m_{lignin}}$$

Yield (%) of ether products is based on the concentration of the β–O–4 linkages:

$$Yield_{ether} = \frac{n_{ether}}{m_{lignin} \times M_{\beta\text{-}O\text{-}4}} \times 100\%$$

n_{ester} was determined by GC based on internal standard curves and areas of integrated peak area.

$M_{\beta\text{–}O\text{–}4}$: molar concentration of β–O–4 linkages per 1 g lignin (mmol/g)

$S_{\beta\text{–O–}4}$: integral area of β–O–4 linkages determained by HSQC

$S_{trioxane}$: integral area of trioxane determained by HSQC

$m_{trioxane}$: weigt of trioxane (mg)

m_{lignin}: weigt of lignin (mg)

M_w (trioxane): formula weight of trioxane

n_{ether}: mol of ether products

Supplementary table and figures

Supplementary Table 1. Summary of recent achievements of oxidative lignin valorization.

Year	Catalyst	Source	T (°C)	Gas	Product(s)	Yield (%)	Mechanistic study	Reference
Homogeneous systems								
2014	$CuSO_4$/phen	Pine MW lignin	80	12 bar O_2	Vanillin, vanillic acid	<10	No	7
2016	Peracetic Acid	Pretreated lignin	60		Phenol mixtures	22	Yes	8
2017	$CuSO_4$	Pine wood	170	3 bar O_2	Vanillin	18	No	9
2018	NaOH	Poplar lignin	175	5 bar O_2 + 15 bar He	Phenol and carboxylic acid mixtures	30	No	10
2020	NaOH	Wood sawdust	160	10 bar O_2	Phenol and carboxylic acid mixtures	20	No	11
2021	Bobbitt's salt	Lignin fraction	100		2,6-dimethoxybenzoquinone	18	Yes	12
Heterogeneous systems								
2018	Au/Li–Al layered double hydroxide	Organosolv lignin	120	1 bar O_2	Phenol and carboxylic acid mixtures	40	Yes	13
2019	Polyoxometalate ionic liquid [BSmim]$CuPW_{12}O_{40}$	Lignin	170	8 bar O_2	Diethyl Maleate	404.8 mg/g	No	14
2019	Polyoxometalate $K_5V_3W_3O_{19}$	Lignin	115	50 bar O_2	Carboxylic acids	<20	No	15
2021	Co-N-C catalyst	Poplar biomass	190	35 bar 6% O_2 in N_2	Phenol mixtures	15	No	16
2021	Polyoxometalate	Wood sawdust	140	10 bar O_2 in N_2 (9:1)	Phenol mixtures	46	No	17

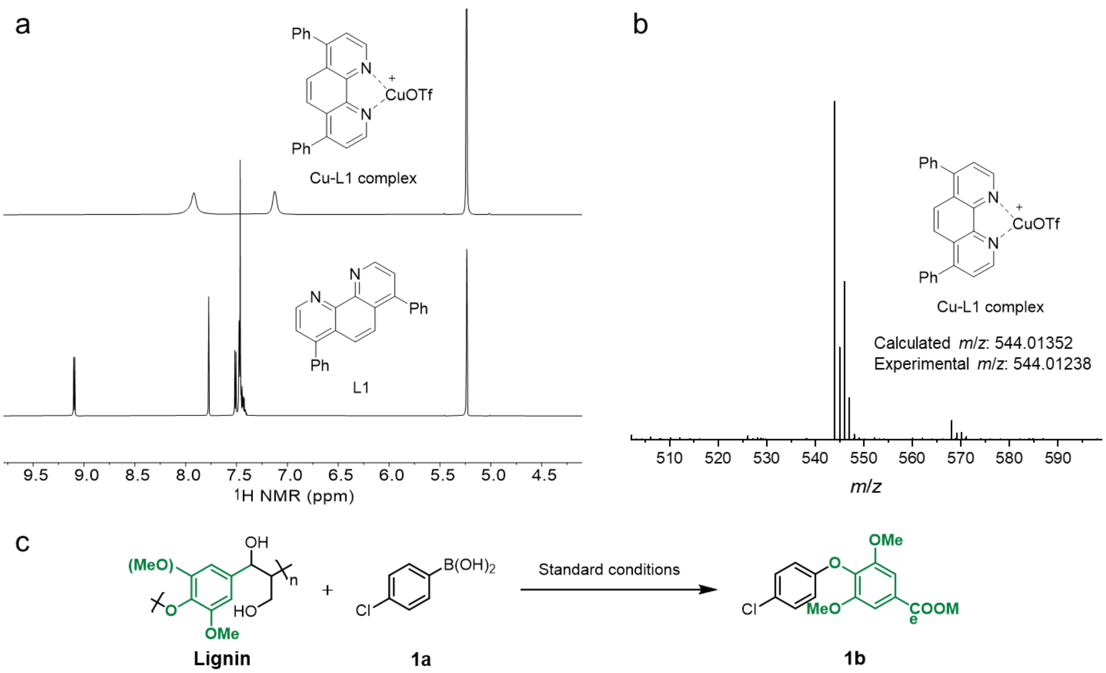

Catalytic system	Yield of **1b** (%)	TOF (h^{-1})
Fresh $Cu(OTf)_2$ and L1	68	51
Recycled $Cu(OTf)_2$-L1 complex	61	49

Supplementary Figure 1. Isolation and reuse of the CuL1 complex. (**a**) ^{1}H NMR spectra of isolated complex and L1 in CD_2Cl_2. (**b**) High resolution electrospray ionization mass spectrometry of the isolated complex. (**c**) Comparison of catalytic efficiency of fresh $Cu(OTf)_2$ and L1 and with the recycled complex.

The complex was isolated by removal of the solvent after reaction and extraction with CH_2Cl_2 (5 mL) and H_2O (5 mL). The organic phase was washed with H_2O (3×5 mL) and concentrated under vacuum. The complex was purified by column chromatography (mobile phase: 10% methanol (volume ratio) in CH_2Cl_2).

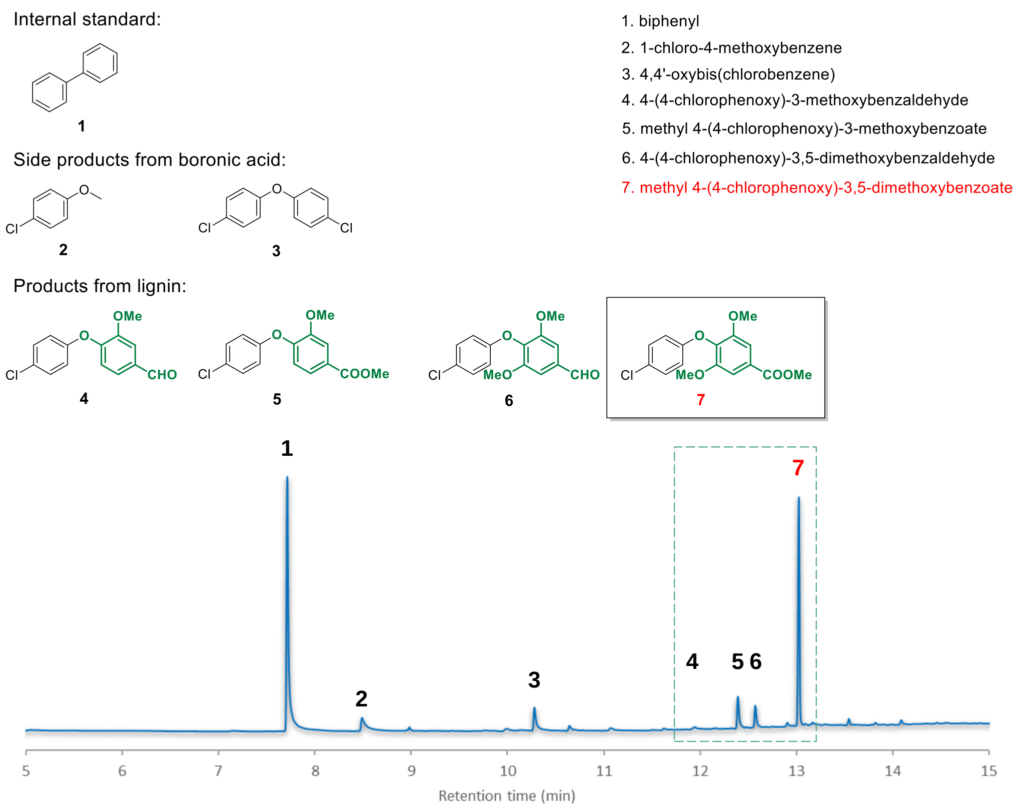

Supplementary Figure 2. Original gas chromatogram of the reaction mixture. Assignment of the main species is shown at the top of the figure.

Beech lignin

Structure	Content (mmol/g lignin)
β-O-4	0.245
α-O-4	0.013
β-β	0.106
S	0.812
G	0.209
H	0

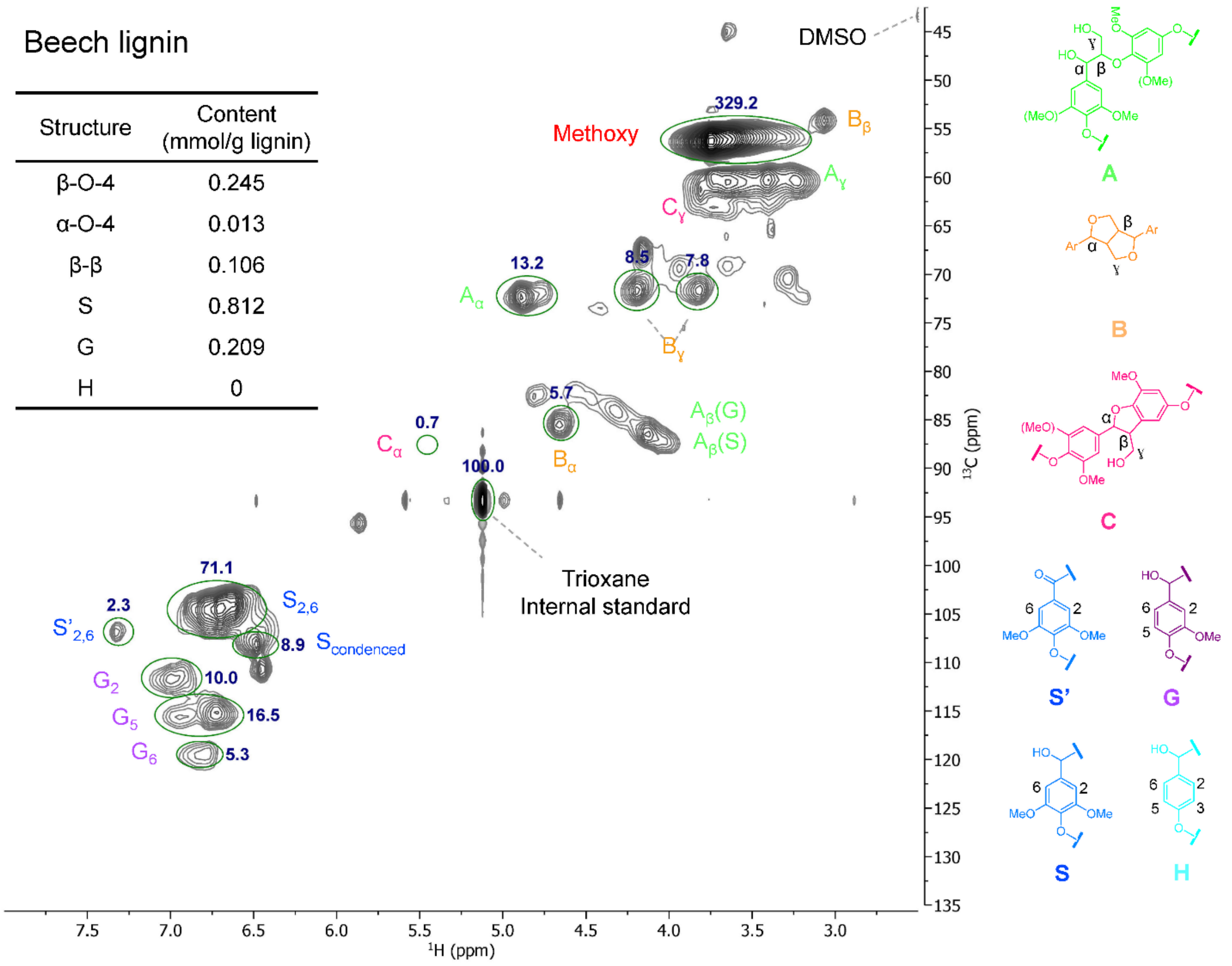

Supplementary Figure 3. 2D HSQC spectrum of isolated **beech** wood lignin before reaction. Contours are color-coded to the corresponding structures. The insert table summaries the occurrence values of bonding motifs and potential linkages of lignin (mmol per 1 g lignin). Trioxane was used as an internal standard to calculate the content of the corresponding structures. 54.6 mg beech lignin and 4.5 mg trioxane was dissolved in 0.5 mL DMSO-d_6.

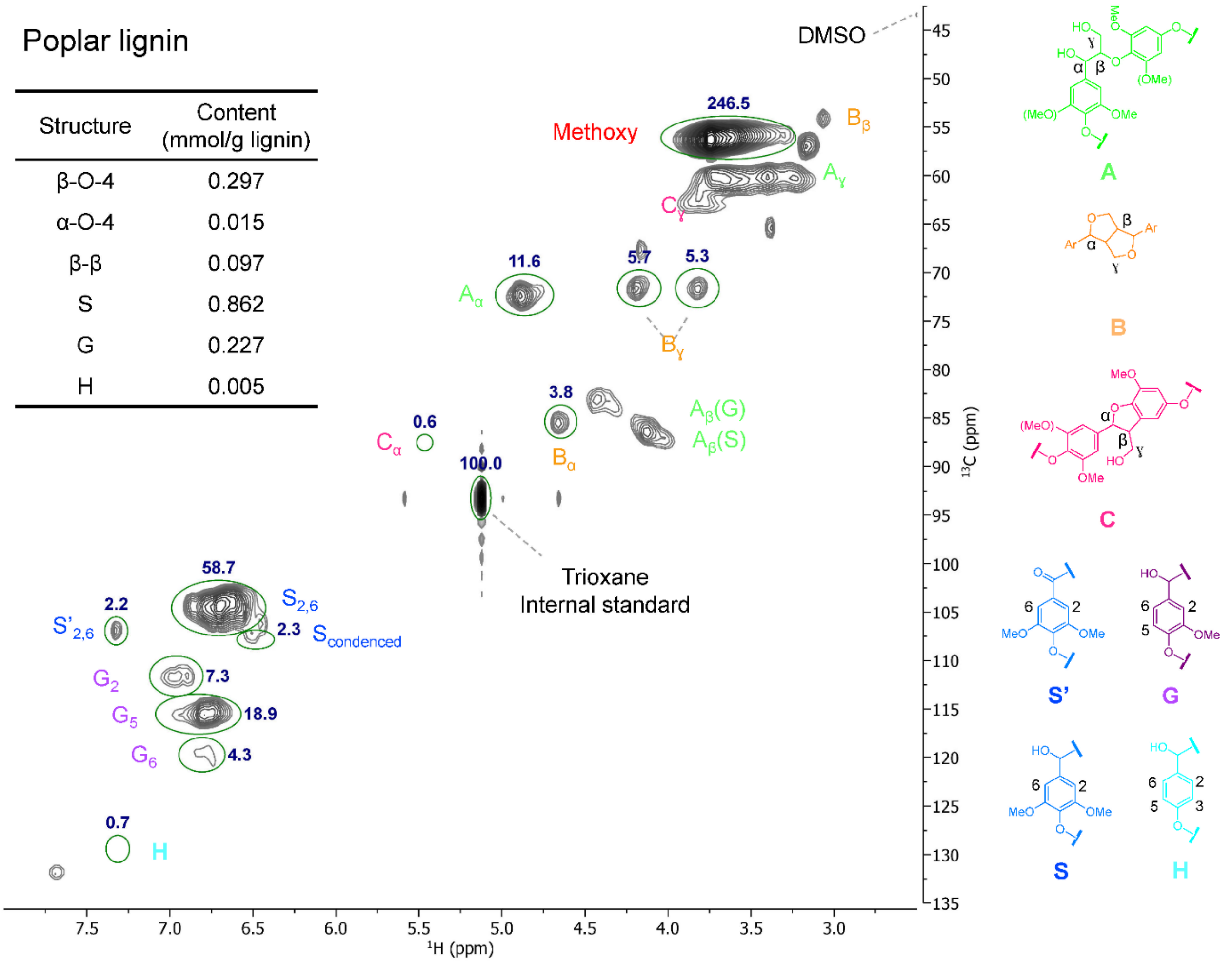

Structure	Content (mmol/g lignin)
β-O-4	0.297
α-O-4	0.015
β-β	0.097
S	0.862
G	0.227
H	0.005

Supplementary Figure 4. 2D HSQC spectrum of isolated **poplar** wood lignin before reaction. Contours are color-coded to the corresponding structures. The insert table summaries the occurrence values of bonding motifs and potential linkages of lignin (mmol per 1 g lignin). Trioxane was used as an internal standard to calculate the content of the corresponding structures. 51.8 mg beech lignin and 5.9 mg trioxane was dissolved in 0.5 mL DMSO-d_6.

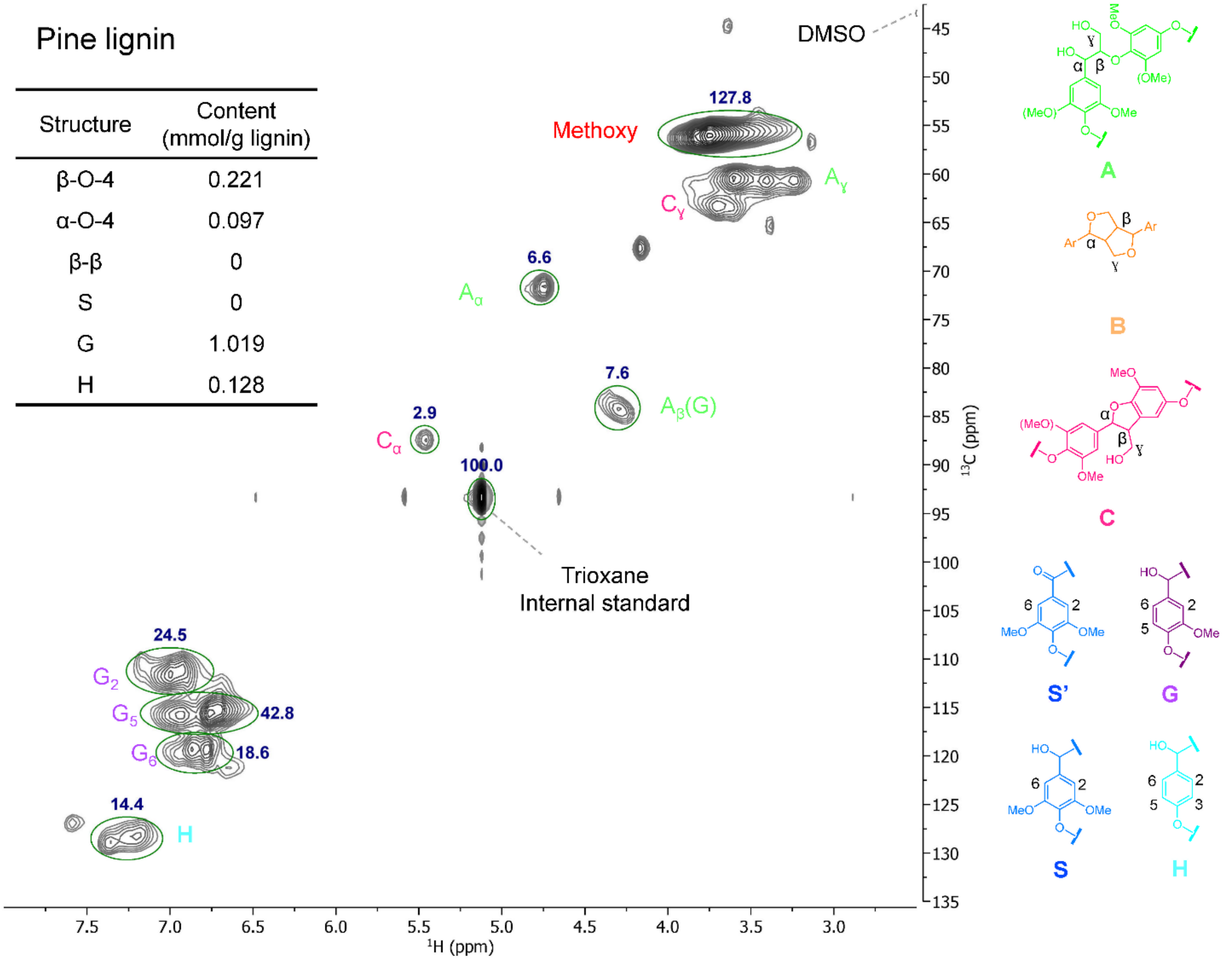

Pine lignin

Structure	Content (mmol/g lignin)
β-O-4	0.221
α-O-4	0.097
β-β	0
S	0
G	1.019
H	0.128

Supplementary Figure 5. 2D HSQC spectrum of isolated **pine** wood lignin before reaction. Contours are color-coded to the corresponding structures. The insert table summaries the occurrence values of bonding motifs and potential linkages of lignin (mmol per 1 g lignin). Trioxane was used as an internal standard to calculate the content of the corresponding structures. 42.4 mg beech lignin and 6.3 mg trioxane was dissolved in 0.5 mL DMSO-d_6.

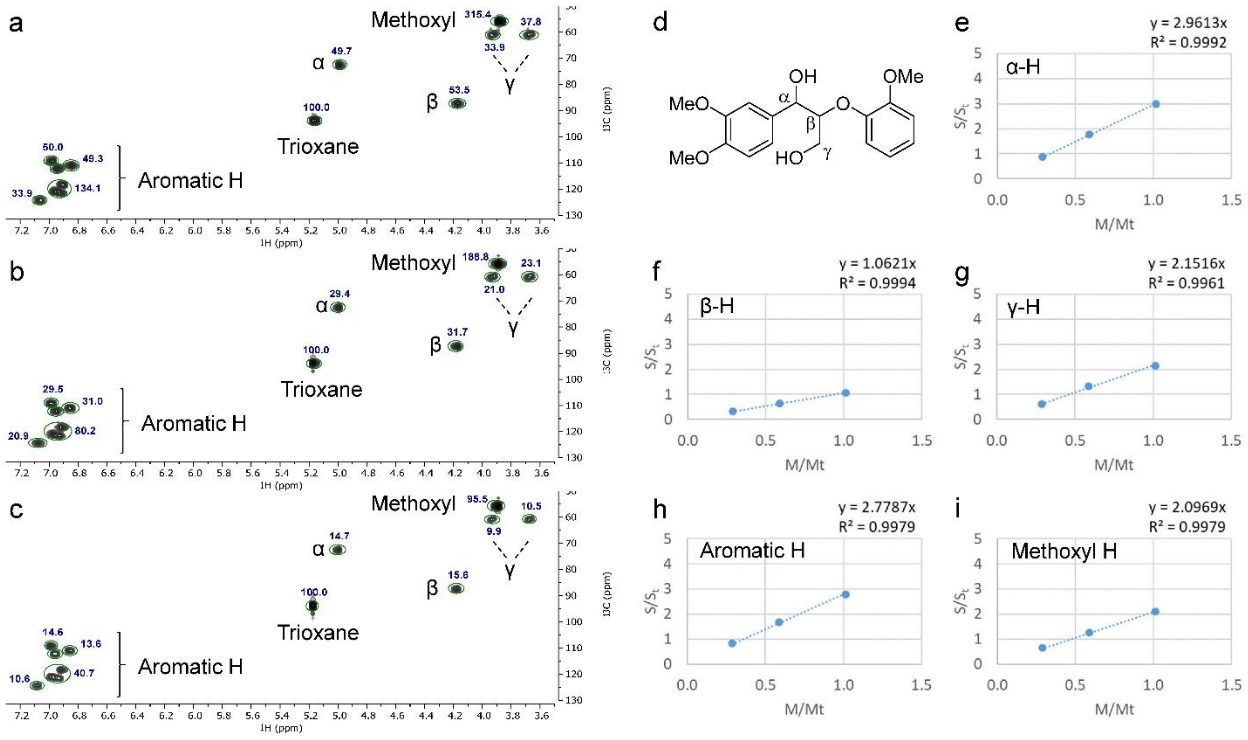

Supplementary Figure 6. Determination of the correction factors for the functional groups in lignin based on HSQC quantitative analysis of the β–O–4 model compound **i**. (**a**) HSQC spectra of **i** (19.2 mg) and internal standard trioxane (5.1 mg) in 0.5 mL DMSO-d_6. (**b**) HSQC spectra of **i** (10.9 mg) and trioxane (5.0 mg) in 0.5 mL DMSO-d_6. (**c**) HSQC spectra of **i** (7.4 mg) and trioxane (6.9 mg) in 0.5 mL DMSO-d_6. (**d**) Structure of β–O–4 model compound **i**. (**e**) Calibration curve of the α-H of **i**. (**f**) Calibration curve of the β-H of **i**. (**g**) Calibration curve of the γ-H of **i**. (**h**) Calibration curve of the aromatic H. (**i**) Calibration curve of the methoxyl H. The slope factor is used as the correction factor. The slope factor (2.9613) of the α-H was used as the correction factor for the β–O–4 linkages.

Supplementary Table 2. Optimization of the Cu catalyst.

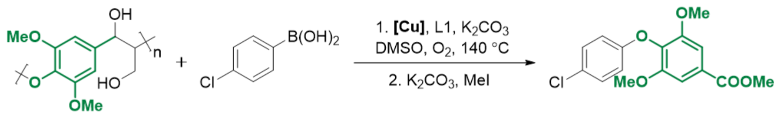

Entry	**[Cu]**	Yield of ether (%)	Yield of **1b** (%)
1	CuO	0	0
2	$CuSO_4$	0	0
3	$Cu(NO_3)_2{\cdot}3H_2O$	83	57
4	$Cu(BF_4)_2$	79	53
**5**	**$Cu(OTf)_2$**	**92**	**68**
6	CuCl	54	42
7	$CuCl_2$	55	36
8	$Cu(OAc)_2$	51	34
9	$Cu(ClO_4)_2$	63	44
10	$Cu(acac)_2$	28	20
11	Copper(II) bis(2-ethylhexanoate)	48	30
12	Copper(II) 2-thiophenecarboxylate	67	45
13	Copper(II) 2-pyrazinecarboxylate	68	47

Reaction conditions: (1) beech lignin (40 mg), 4-chlorophenylboronic acid (1.5 equiv.), **Cu salt** (30 mol%), bathophenanthroline (L1, 30 mol%), K_2CO_3 (4 equiv.), biphenyl (0.02 mmol), DMSO (2 mL), O_2 (3 atm), 140 ºC, 6 h. (2) K_2CO_3 (3 equiv.), MeI (10 equiv.), 25 ºC, 12 h.

Supplementary Table 3. Optimization of the base.

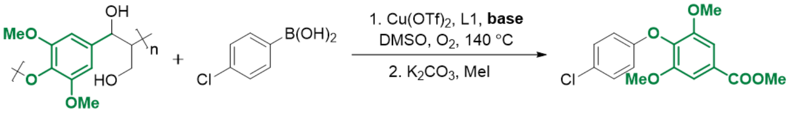

Beech lignin   **1a**   **1b**

Entry	Base	Yield of ether (%)	Yield of **1b** (%)
1	Cs_2CO_3	81	59
**2**	**K_2CO_3**	**92**	**68**
3	Na_2CO_3	83	50
4	Li_2CO_3	69	37
5	NaOH	93	61
6	KOH	90	55
7	K_3PO_4	71	40
8	$(CH_3)_3COK$	47	34
9	CH_3CH_2ONa	71	40
10	CH_3ONa	76	42

Reaction conditions: (1) beech lignin (40 mg), 4-chlorophenylboronic acid (1.5 equiv.), $Cu(OTf)_2$ (30 mol%), L1 (30 mol%), **base** (4 equiv.), biphenyl (0.02 mmol), DMSO (2 mL), O_2 (3 atm), 140 ºC, 6 h. (2) K_2CO_3 (3 equiv.), MeI (10 equiv.), 25 ºC, 12 h.

Supplementary Table 4. Optimization of the co-catalyst (ligand).

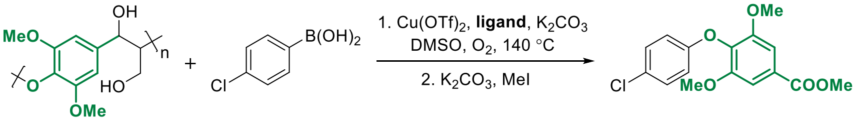

Beech lignin   **1a**   **1b**

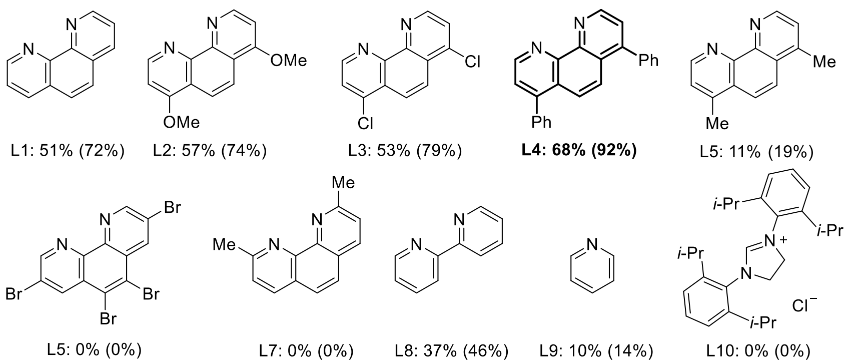

Yield of all ethers is listed in the brackets. Reaction conditions: (1) beech lignin (40 mg), 4-chlorophenylboronic acid (1.5 equiv.), $Cu(OTf)_2$ (30 mol%), **ligand** (30 mol%), K_2CO_3 (4 equiv.), biphenyl (0.02 mmol), DMSO (2 mL), O_2 (3 atm), 140 ºC, 6 h. (2) K_2CO_3 (3 equiv.), MeI (10 equiv.), 25 ºC, 12 h.

Supplementary Table 5. Optimization of the solvent.

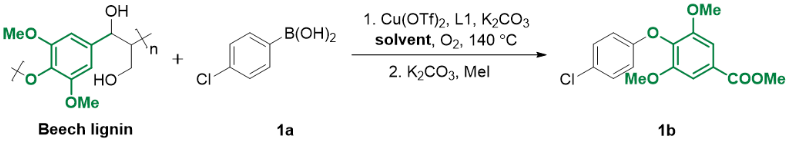

Entry	**Solvent**	Yield of ether (%)	Yield of **1b** (%)
1	H_2O	0	0
2	Dimethylformamide	52	35
3	**Dimethyl sulfoxide**	**92**	**68**
4	N-methylpyrrolidone	47	28
5	MeOH	0	0
6	CH_3CN	19	11
7	Dioxane	0	0
8	CH_2Cl_2	0	0

Reaction conditions: (1) beech lignin (40 mg), 4-chlorophenylboronic acid (1.5 equiv.), $Cu(OTf)_2$ (30 mol%), L1 (30 mol%), K_2CO_3 (4 equiv.), biphenyl (0.02 mmol), **solvent** (2 mL), O_2 (3 atm), 140 ºC, 6 h. (2) K_2CO_3 (3 equiv.), MeI (10 equiv.), 25 ºC, 12 h.

Supplementary Table 6. Optimization of the amount of catalyst and ligand.

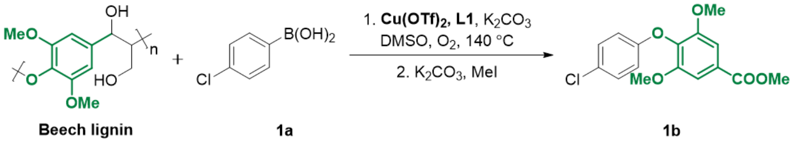

Entry	**$Cu(OTf)_2$**	**L1**	Yield of ether (%)	Yield of **1b** (%)
1	10 mol%	10 mol%	5	4
2	15 mol%	15 mol%	15	13
**3**	**30 mol%**	**30 mol%**	**92**	**68**
4	60 mol%	60 mol%	84	57
5	100 mol%	100 mol%	76	44
6	30 mol%	0	41	29
7	30 mol%	15 mol%	71	55
8	30 mol%	80 mol%	65	42

Reaction conditions: (1) beech lignin (40 mg), 4-chlorophenylboronic acid (1.5 equiv.), $Cu(OTf)_2$ (**15-100 mol%**), L1 (**0-100 mol%**), K_2CO_3 (4 equiv.), biphenyl (0.02 mmol), DMSO (2 mL), O_2 (3 atm), 140 ºC, 6 h. (2) K_2CO_3 (3 equiv.), MeI (10 equiv.), 25 ºC, 12 h.

Supplementary Table 7. Optimization of the amount of base.

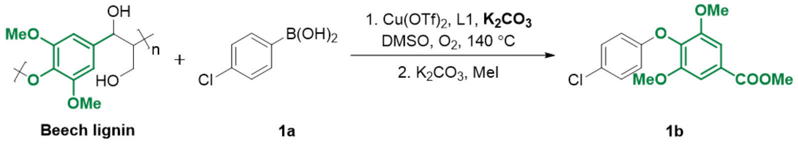

Entry	K_2CO_3	Yield of ether (%)	Yield of **1b** (%)
1	0 equiv.	0	0
2	1 equiv.	51	28
3	2 equiv.	59	41
4	3 equiv.	77	55
**5**	**4 equiv.**	**92**	**68**
6	5 equiv.	83	60
7	6 equiv.	54	33

Reaction conditions: (1) beech lignin (40 mg), 4-chlorophenylboronic acid (1.5 equiv.), $Cu(OTf)_2$ (30 mol%), L1 (30 mol%), K_2CO_3 (**0-6 equiv.**), biphenyl (0.02 mmol), DMSO (2 mL), O_2 (3 atm), 140 ºC, 6 h. (2) K_2CO_3 (3 equiv.), MeI (10 equiv.), 25 ºC, 12 h.

Supplementary Table 8. Optimization of the atmosphere.

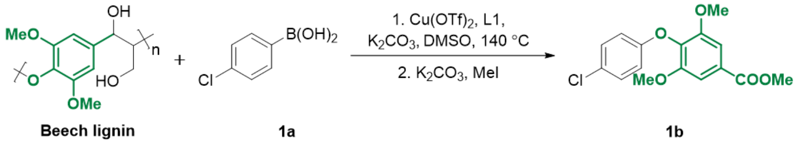

Entry	**Atmosphere**	Yield of ether (%)	Yield of **1b** (%)
1	1 atm O_2	68	46
**2**	**3 atm O_2**	**92**	**68**
3	1 atm air	7	3
4	10 atm air	85	56
5	1 atm N_2	0	0

Reaction conditions: (1) beech lignin (40 mg), 4-chlorophenylboronic acid (1.5 equiv.), $Cu(OTf)_2$ (30 mol%), L1 (30 mol%), K_2CO_3 (4 equiv.), biphenyl (0.02 mmol), DMSO (2 mL), air/O_2/N_2, 140 ºC, 6 h. (2) K_2CO_3 (3 equiv.), MeI (10 equiv.), 25 ºC, 12 h.

Supplementary Table 9. Optimization of the reaction time.

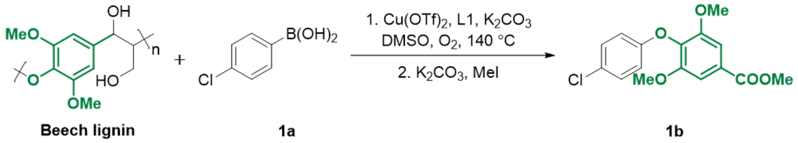

Entry	**Reaction time (h)**	Yield of ether (%)	Yield of **1b** (%)
1	2	61	36
2	4	82	51
**3**	**6**	**92**	**68**
4	8	94	67
5	10	92	68

Reaction conditions: (1) beech lignin (40 mg), 4-chlorophenylboronic acid (1.5 equiv.), $Cu(OTf)_2$ (30 mol%), L1 (30 mol%), K_2CO_3 (4 equiv.), biphenyl (0.02 mmol), DMSO (2 mL), O_2 (3 atm), 140 ºC, **2-10 h**. (2) K_2CO_3 (3 equiv.), MeI (10 equiv.), 25 ºC, 12 h.

Supplementary Table 10. Optimization of the reaction temperature.

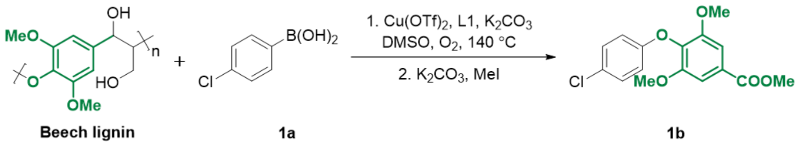

Entry	**Reaction temperature (ºC)**	Yield of ether (%)	Yield of **1b** (%)
1	100	12	4
2	120	73	51
3	130	83	54
**4**	**140**	**92**	**68**
5	150	92	65

Reaction conditions: (1) beech lignin (40 mg), 4-chlorophenylboronic acid (1.5 equiv.), $Cu(OTf)_2$ (30 mol%), L1 (30 mol%), K_2CO_3 (4 equiv.), biphenyl (0.02 mmol), DMSO (2 mL), O_2 (3 atm), **100-140 ºC**, 6 h. (2) K_2CO_3 (3 equiv.), MeI (10 equiv.), 25 ºC, 12 h.

Supplementary Table 11. Optimization of solvent for the methylation step.

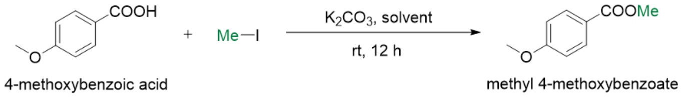

Entry	**Solvent**	Yield of ester (%)
1	DMF	>99
2	DMSO	>99
3	Ethyl acetate	4
4	Acetonitrile	18
5	THF	29

Reaction conditions: carboxylic acid (1 mmol), K_2CO_3 (3 equiv.), MeI (10 equiv.), **solvent** (5 mL), 25 ºC, 12 h.

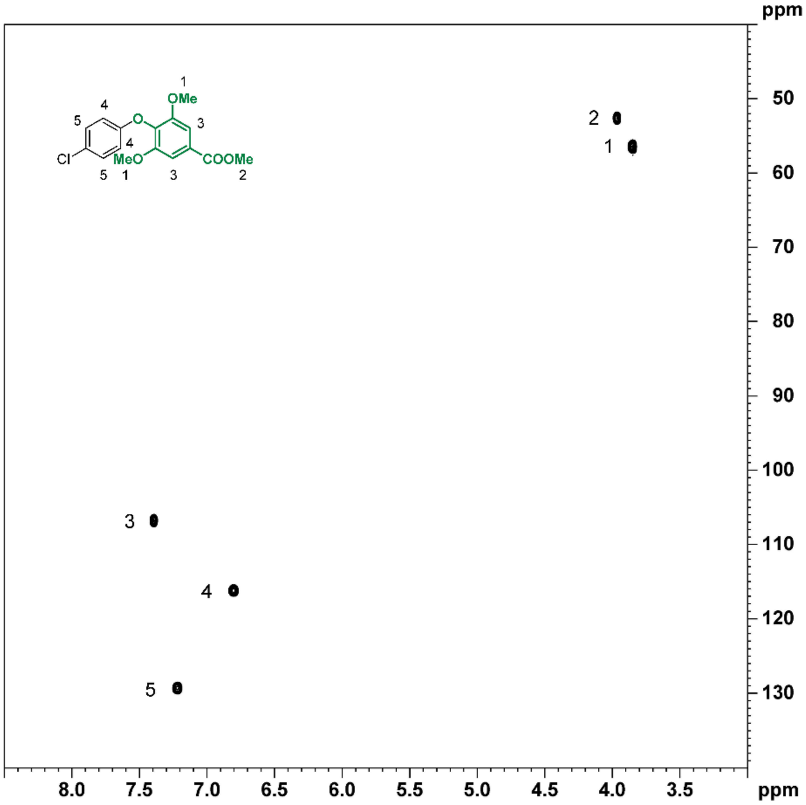

Supplementary Figure 7. HSQC spectrum of methyl 4-(4-chlorophenoxy)-3,5-dimethoxybenzoate **1a**. Assignment of contours may be appreciated from the structure (top, left).

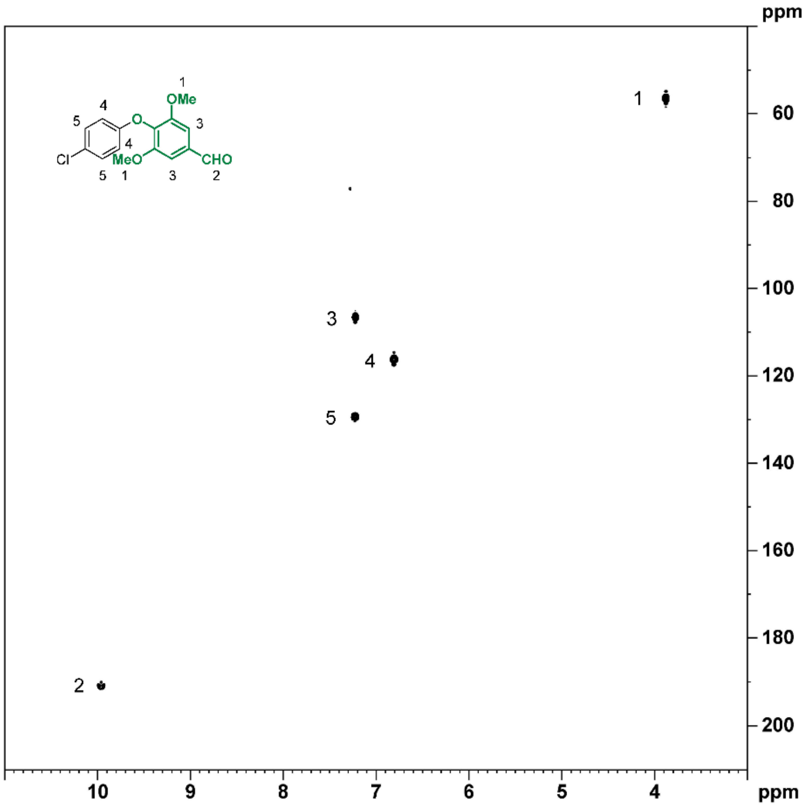

Supplementary Figure 8. HSQC spectrum of 4-(4-chlorophenoxy)-3,5-dimethoxybenzaldehyde **2a**. Assignment of contours may be appreciated from the structure (top, left).

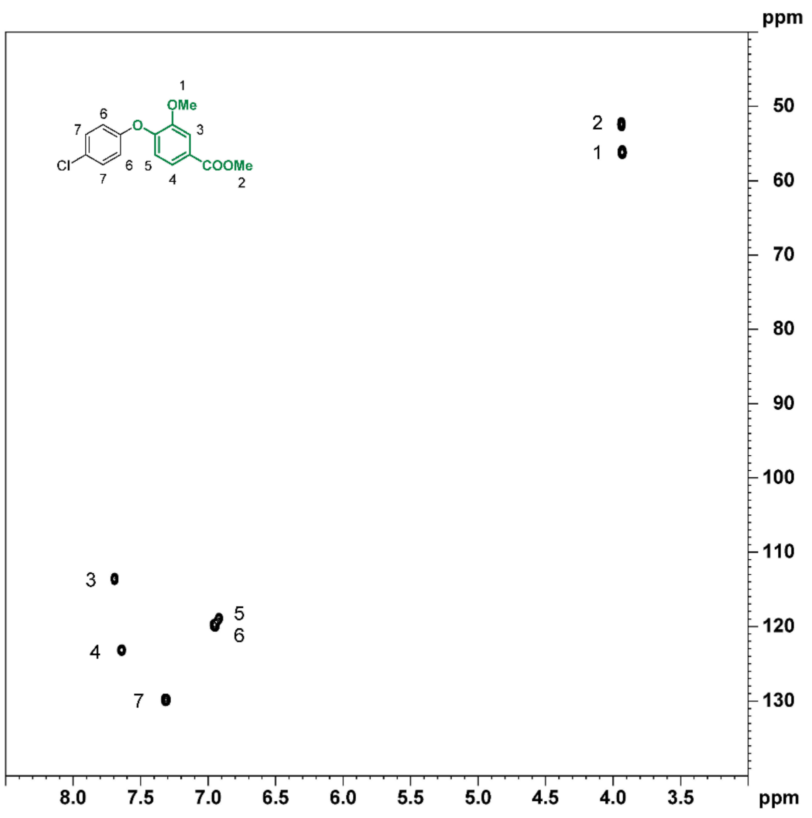

Supplementary Figure 9. HSQC spectrum of methyl 4-(4-chlorophenoxy)-3-methoxybenzoate **3a**. Assignment of contours may be appreciated from the structure (top, left).

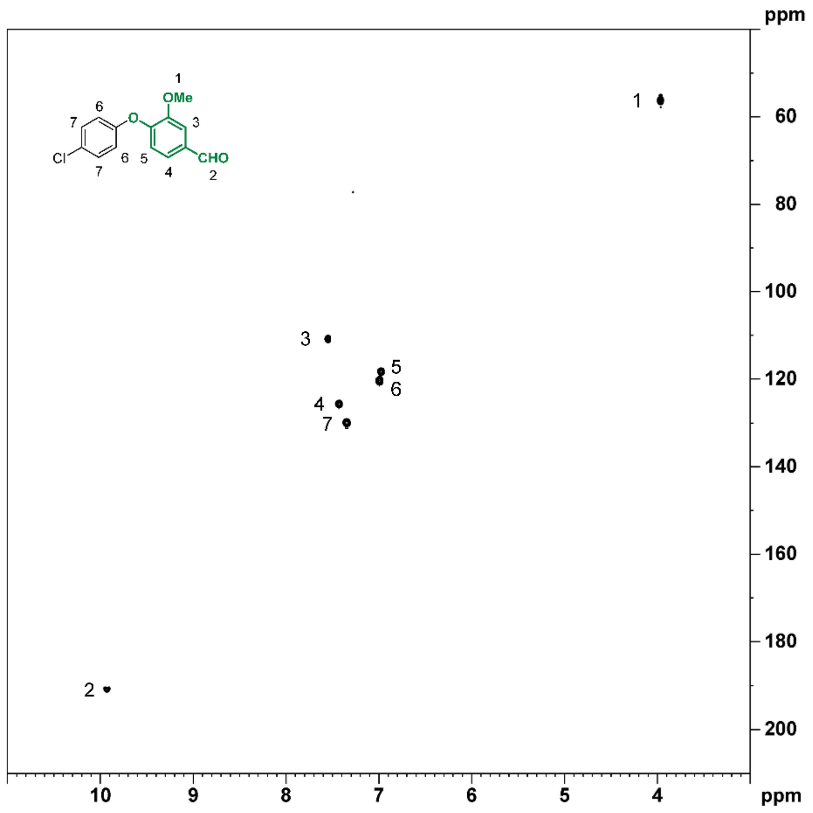

Supplementary Figure 10. HSQC spectrum of 4-(4-chlorophenoxy)-3-methoxybenzaldehyde **4a**. Assignment of contours may be appreciated from the structure (top, left).

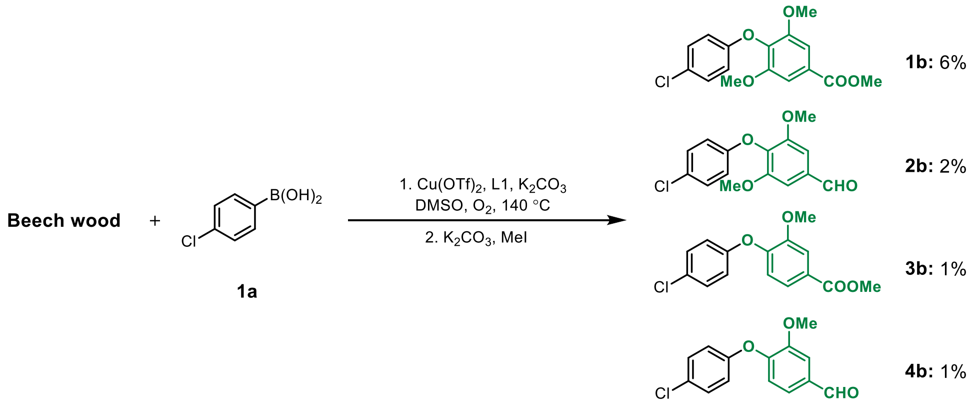

Supplementary Figure 11. Application of beech wood sawdust as the lignin source. Reaction conditions: (1) Beech wood (100 mg), 4-chlorophenylboronic acid (1.5 equiv.), $Cu(OTf)_2$ (30 mol%), L1 (30 mol%), K_2CO_3 (4 equiv.), biphenyl (0.02 mmol, internal standard), DMSO (2 mL), O_2 (3 atm), 140 ºC, 6 h. (2) K_2CO_3 (3 equiv.), MeI (10 equiv.), 25 ºC, 12 h.

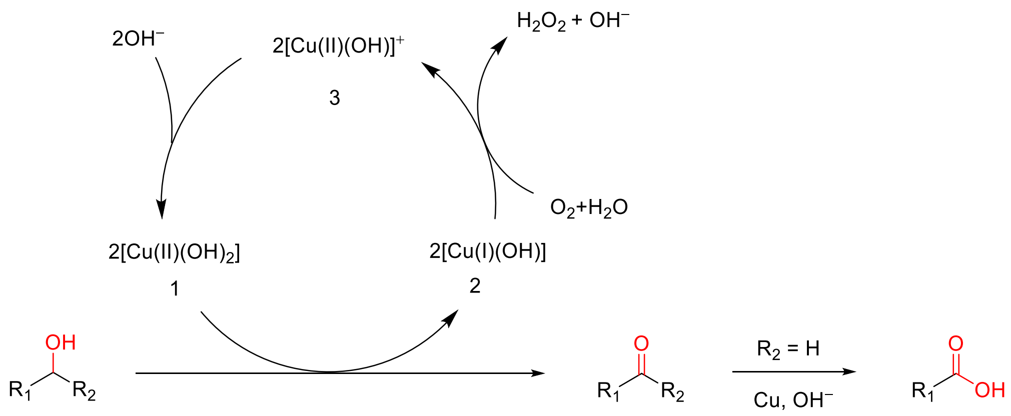

Supplementary Figure 12. Proposed reaction mechanism for the Cu catalyzed aerobic oxidation of alcohols under basic reaction conditions.

Oxidation of alcohol by Cu(II) species **1** gives and aldehyde or ketone with concommitant reduction of the catalyst to Cu(I) species **2**, which are subsequently transformed to **1** via intermediate **3** following reaction with O_2 and consumption of OH^-.[18] If aldehyde is genereated, oxidation of the aldehyde afords a carboxylic acid under the basic reaction conditions.[19]

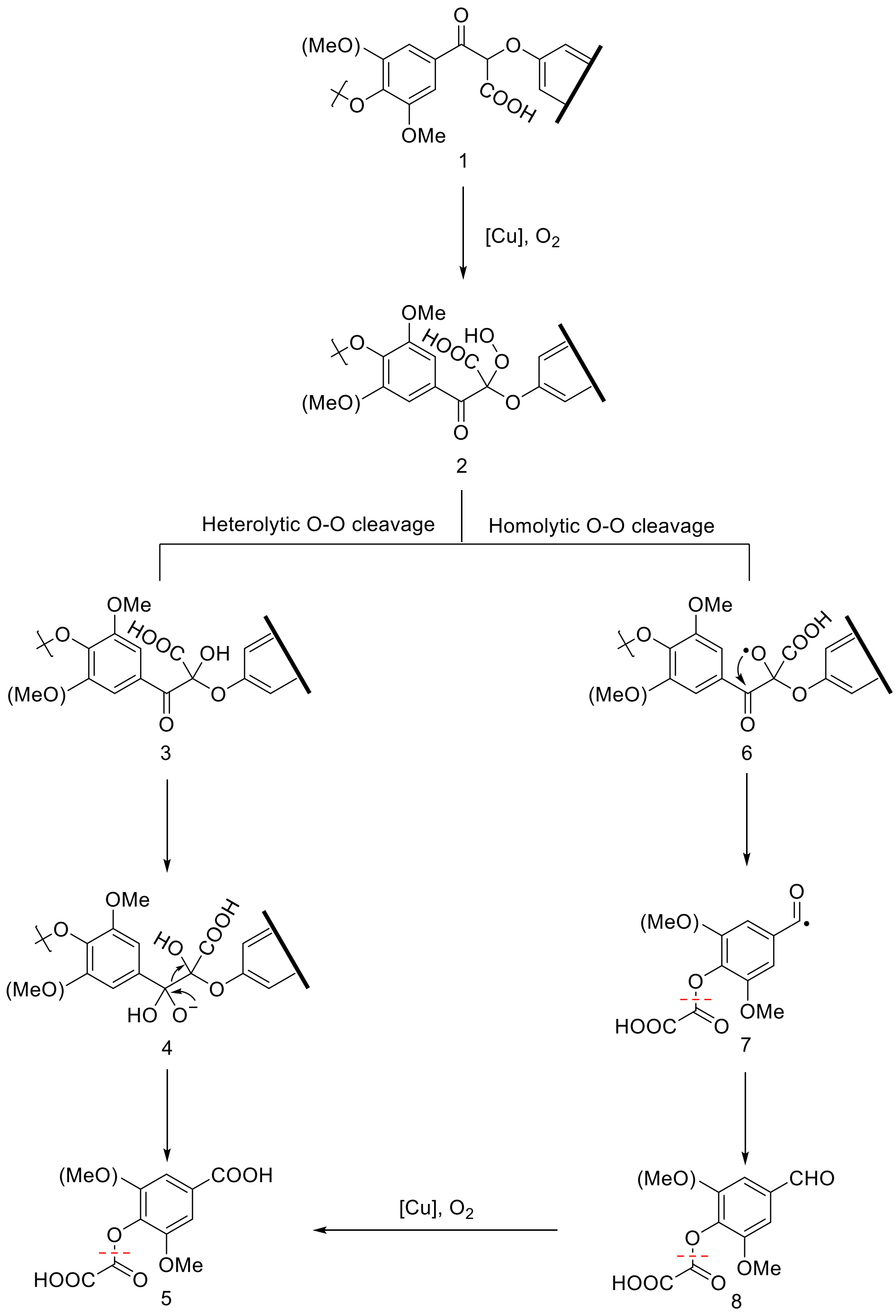

Supplementary Figure 13. Proposed reaction mechanism for the Cu catalyzed cleavage of C_α–C_β bonds.

The carbonyl intermediate **1** is activated and oxygenated under Cu catalyzed basic reaction conditions, resulting in the formation of organic peroxide intermediate **2**.[20-22] Heterolytic or homolytic cleavage of the O–O bond leads to two possible reaction pathways. (1) Heterolytic cleavage of organic peroxide forms hydroxy compound **3**. Cu promoted nucleophilic of OH^- to carbonyl group of **3** and subsequent C–C bond scission of **4** will give oxalic acid protected phenol intermediate **5** with a carboxylic acid group. (2) Homolytic cleavage of the O–O bond in **2** will generate α-oxy radical **6**, which will initiate C–C bond scission to form benzoyl radical **7**, followed by oxidation to oxalic acid protected phenol intermediate **8** with an aldehyde group. Oxidation of aldehyde **8** also will produce carboxylic acid **5**. According to the product

distribution (Fig. 2b, carboxylic acid products are the main products with little aldehyde by-products), the two reaction pathways for C_α–C_β bond cleavage both take place.

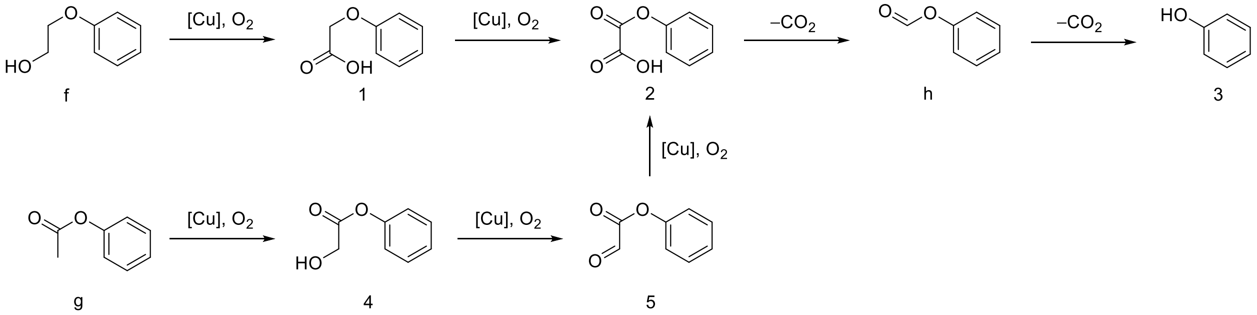

Supplementary Figure 14. Proposed reaction mechanism for the Cu catalyzed deprotection of protected phenols.

For glycol protected phenol **f**, oxidation of the hydroxyl group affords carboxylic acid **1**, which triggers the oxidation of the H-atom on the β-C to form oxalate phenol ester **2** (same as Supplementary Fig. 13, step 1). Following thermal decomposition reactive phenol intermediates would be released, which may be captured through Chan-Lam cross coupling with boronic acids.[23-24] For acetic acid protected phenol **g**, the carbonyl group of **g** could also promote the stepwise oxidation of the H-atom on the α-C to form oxalate phenol ester **2**, followed by thermal decomposition to release phenol.

a

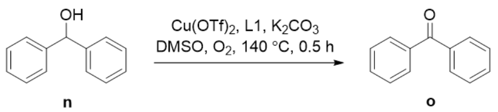

Entry	[Cu]	Ligand	Base	Yield of **o** (%)
1	$Cu(OTf)_2$	L1	K_2CO_3	>99
2		L1	K_2CO_3	3
3	$Cu(OTf)_2$		K_2CO_3	98
4	$Cu(OTf)_2$	L1		2

b

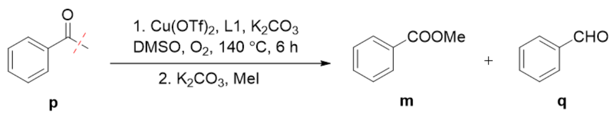

Entry	[Cu]	Ligand	Base	Yield of **m** (%)	Yield of **q** (%)
1	$Cu(OTf)_2$	L1	K_2CO_3	56	40
2		L1	K_2CO_3	0	0
3	$Cu(OTf)_2$		K_2CO_3	90	1
4	$Cu(OTf)_2$	L1		0	0

c

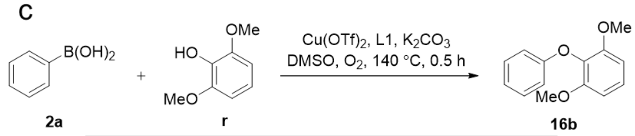

Entry	[Cu]	Ligand	Base	Yield of **r** (%)
1	$Cu(OTf)_2$	L1	K_2CO_3	>99
2		L1	K_2CO_3	0
3	$Cu(OTf)_2$		K_2CO_3	21
4	$Cu(OTf)_2$	L1		40

d

Summary of functions of each compound

Reaction	$Cu(OTf)_2$	L1	K_2CO_3
Alcohol 1 to ketone 2 (−OH group oxidation)	√		√
Ketone 2 to acid 4 (C−C bond activation)	√		√
Acid 4 to diaryl ether 5 (C−O bond formation)	√	√	√

Supplementary Figure 15. Control experiments. (**a**) Catalytic oxidation of 1,1-diphenylmethanol **n** to benzophenone **o**. (**b**) Catalytic C−C bond activation of acetophenone **p** to afford methyl benzoate **m** and benzaldehyde **q**. (**c**) Catalytic cross coupling of phenylboronic acid **2a** and 2,6-dimethylphenol **r**. (**d**) Summary of the functions of $Cu(OTf)_2$, L1 and K_2CO_3.

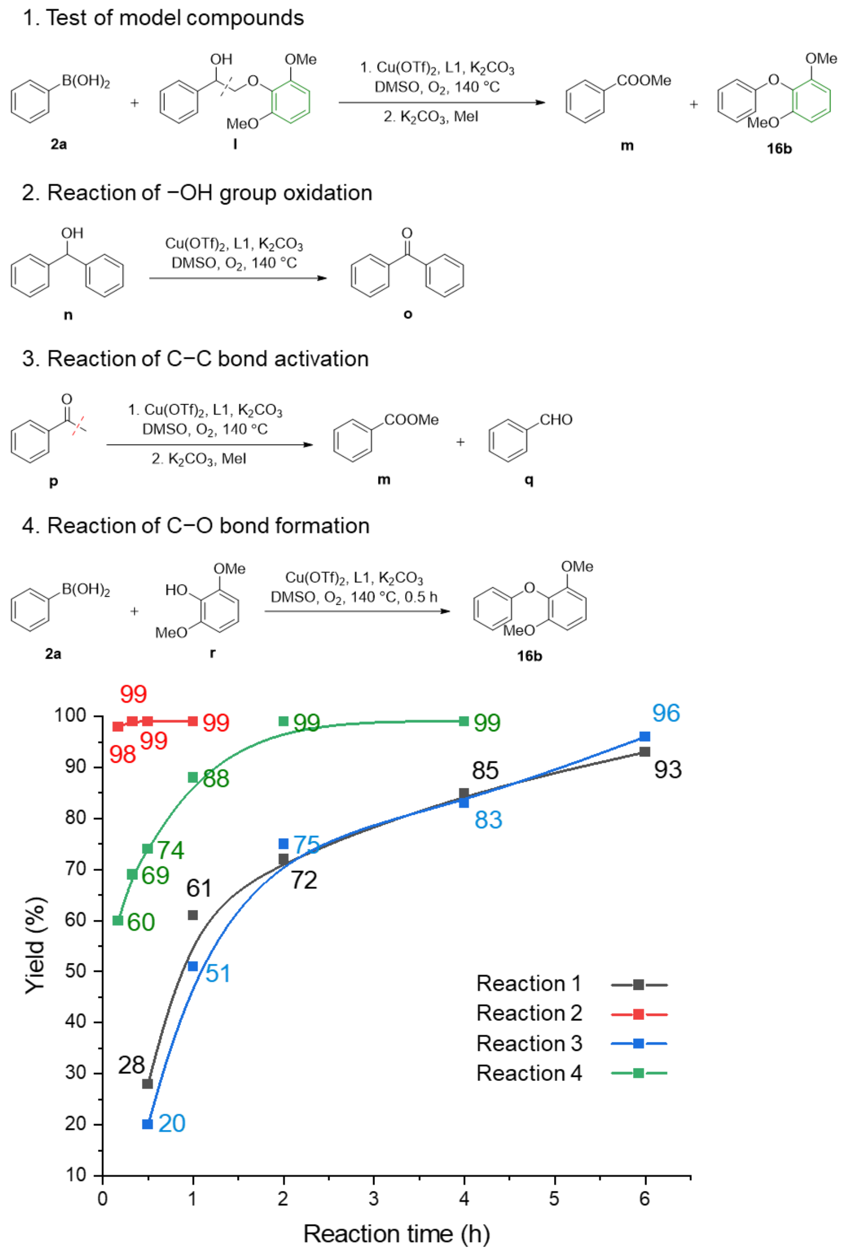

Supplementary Figure 16. Kinetic study. Reaction 1 corresponds to the cross-coupling reaction between boronic acid **2a** and lignin model compound **l** to afford diaryl ether **16b** and byproduct **m** (combination of −OH group oxidation, C−C bond activation and Cham-Lam coupling). Reaction 2 is the oxidation of 1,1-diphenylmethanol **n** to benzophenone **o** (−OH group oxidation). Reaction 3 is the oxidation of acetophenone **p** to methyl benzoate **m** and benzaldehyde **q** (C−C bond activation). Reaction conditions are the same as those reported in Supplementary Fig. 15. Reaction 4 corresponds to the coupling of phenylboronic acid **2a** and 2,6-dimethylphenol **r** to afford diaryl ether **16b** (Cham-Lam coupling). The order of reaction rate is: reaction 2 > reaction 4 > reaction 3 ≈ reaction 1.

Supplementary NMR spectra

NMR spectra of substrates:

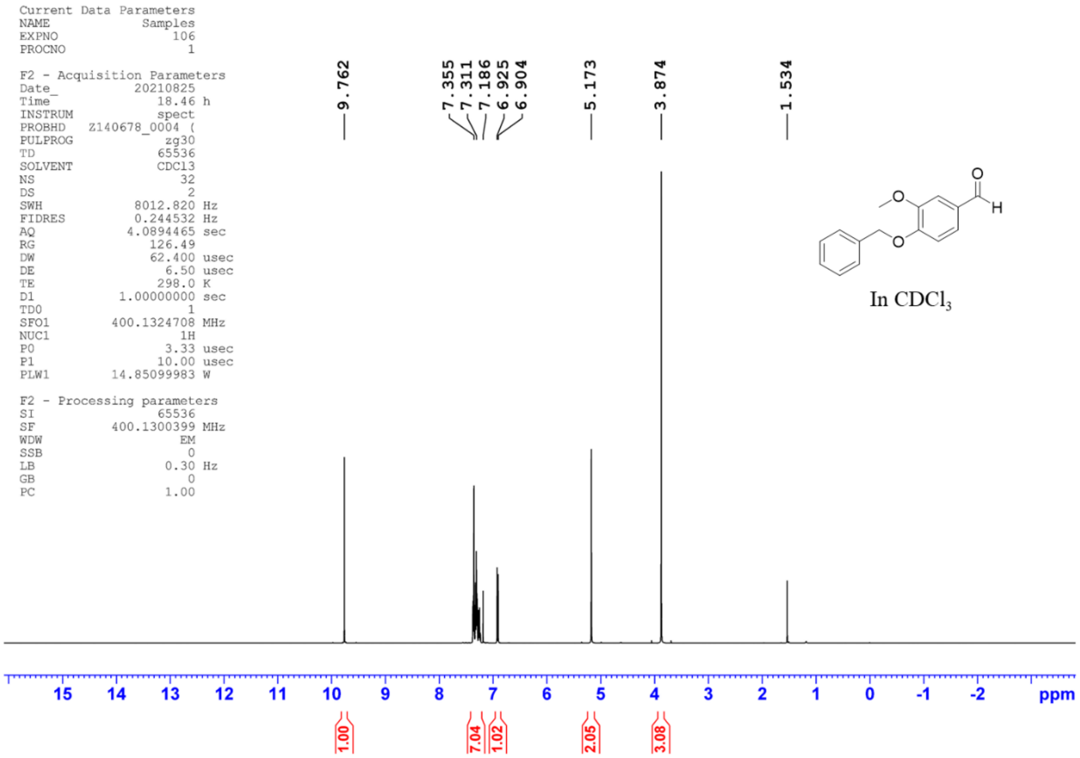

Supplementary Figure 17. ^{1}H NMR spectra of compound 3-methoxy-4-(phenylmethoxy)benzaldehyde.

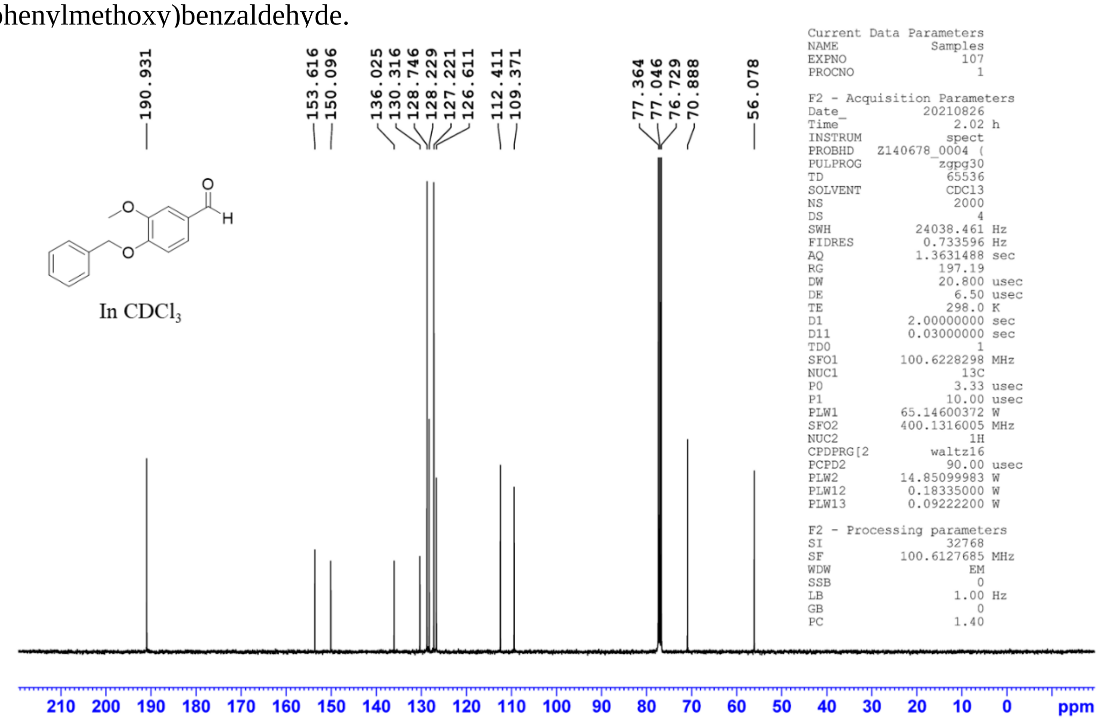

Supplementary Figure 18. ^{13}C NMR spectra of compound 3-methoxy-4-(phenylmethoxy)benzaldehyde.

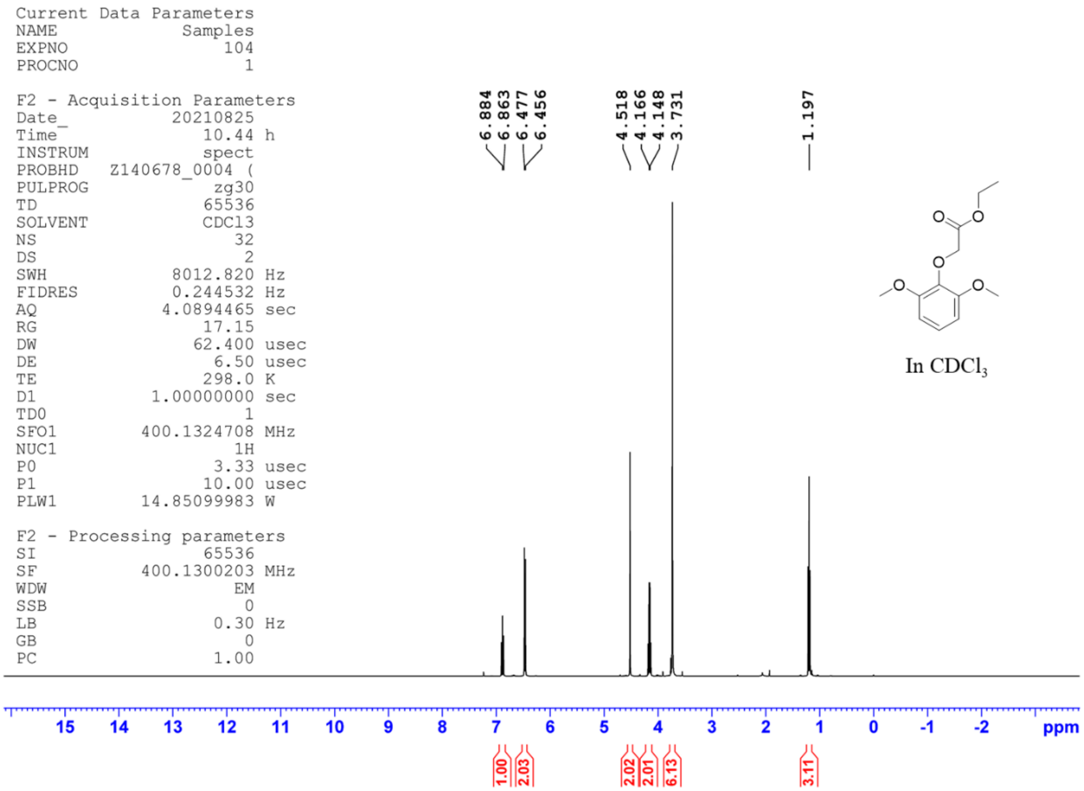

Supplementary Figure 19. ^{1}H NMR spectra of compound ethyl 2-(2,6-dimethoxyphenoxy)acetate.

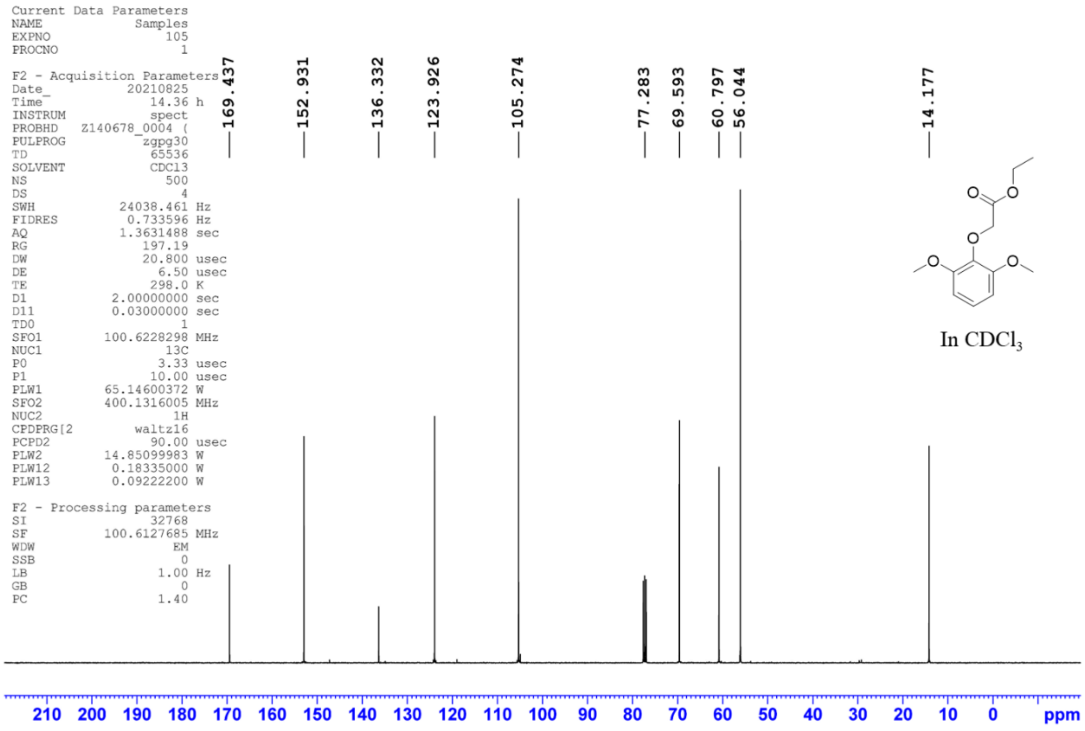

Supplementary Figure 20. ^{13}C NMR spectra of compound ethyl 2-(2,6-dimethoxyphenoxy)acetate.

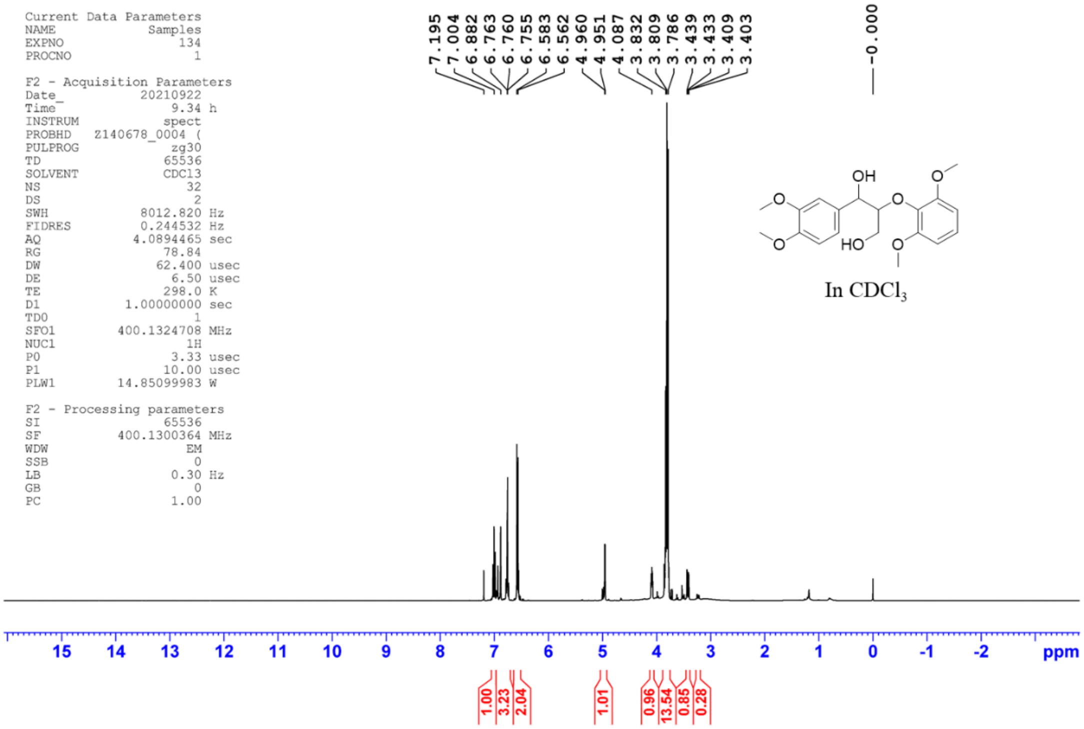

Supplementary Figure 21. ^{1}H NMR spectra of compound **i**.

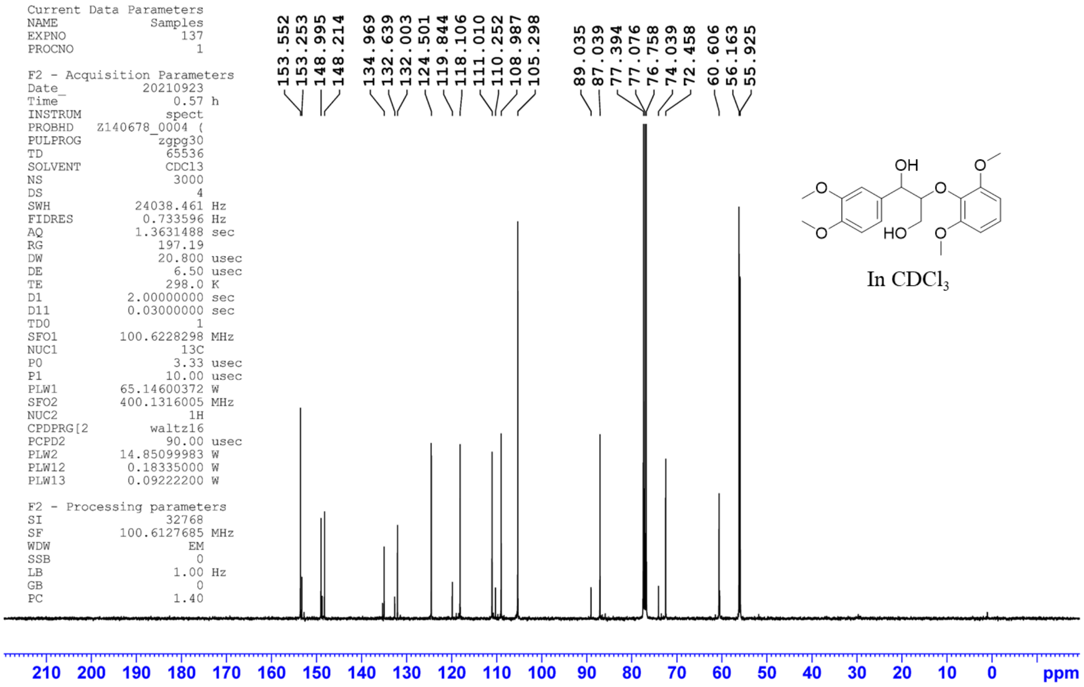

Supplementary Figure 22. ^{13}C NMR spectra of compound **i**.

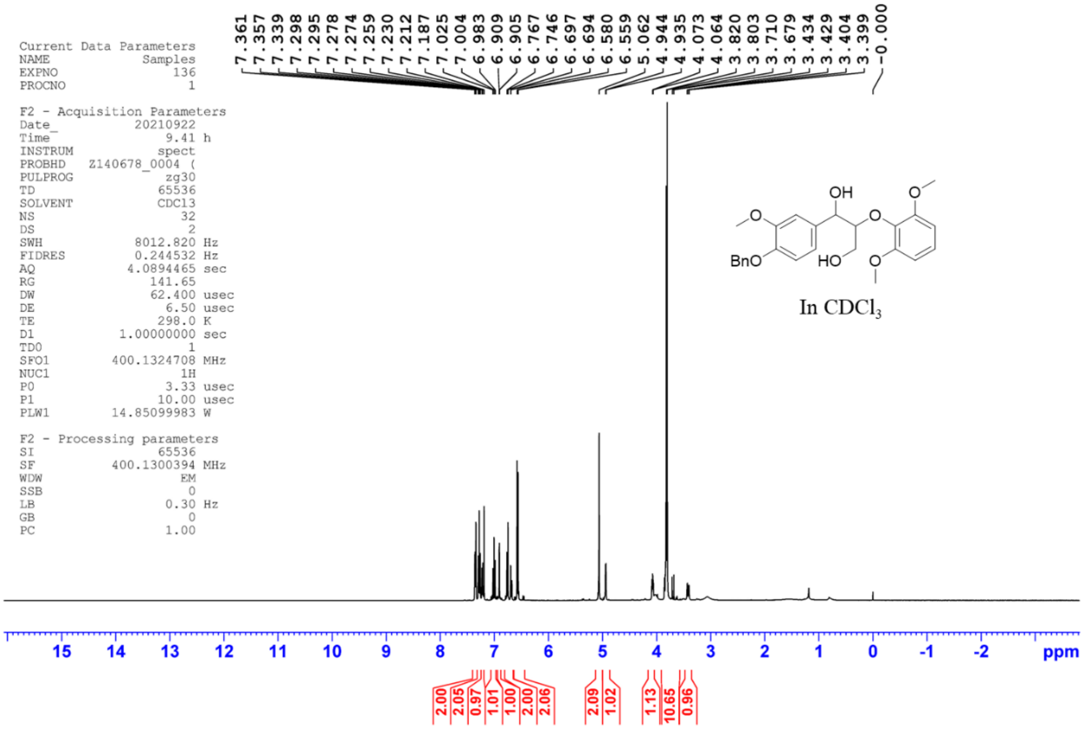

Supplementary Figure 23. ^{1}H NMR spectra of compound 1-(4-(benzyloxy)-3-methoxyphenyl)-2-(2,6-dimethoxyphenoxy)propane-1,3-diol.

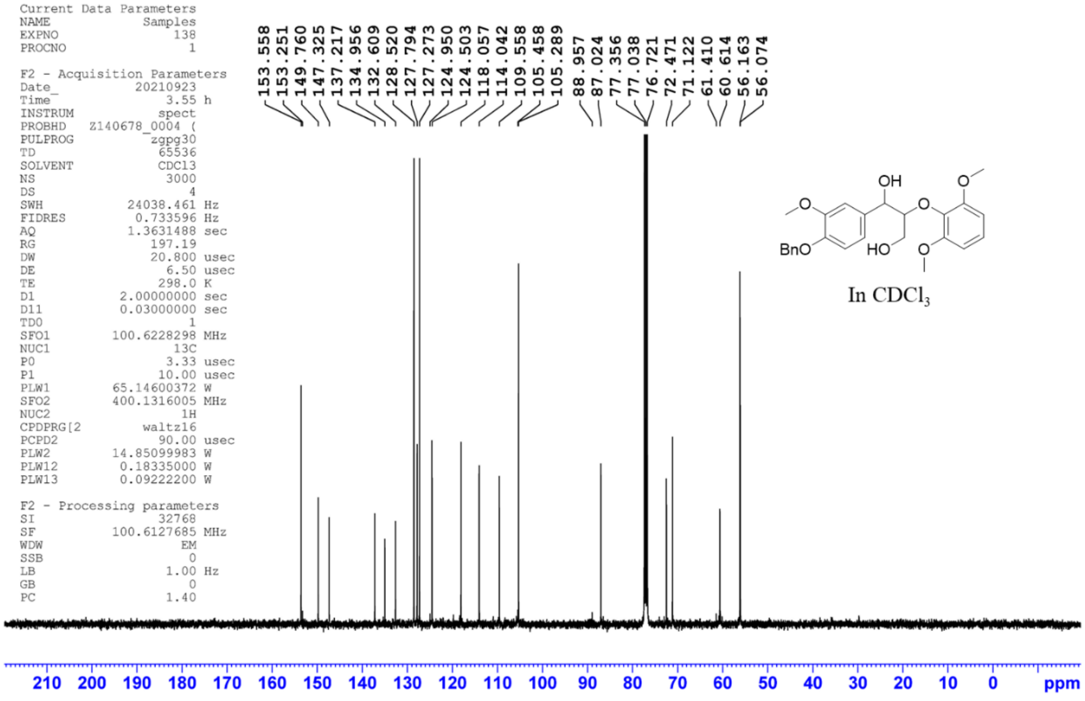

Supplementary Figure 24. ^{13}C NMR spectra of compound 1-(4-(benzyloxy)-3-methoxyphenyl)-2-(2,6-dimethoxyphenoxy)propane-1,3-diol.

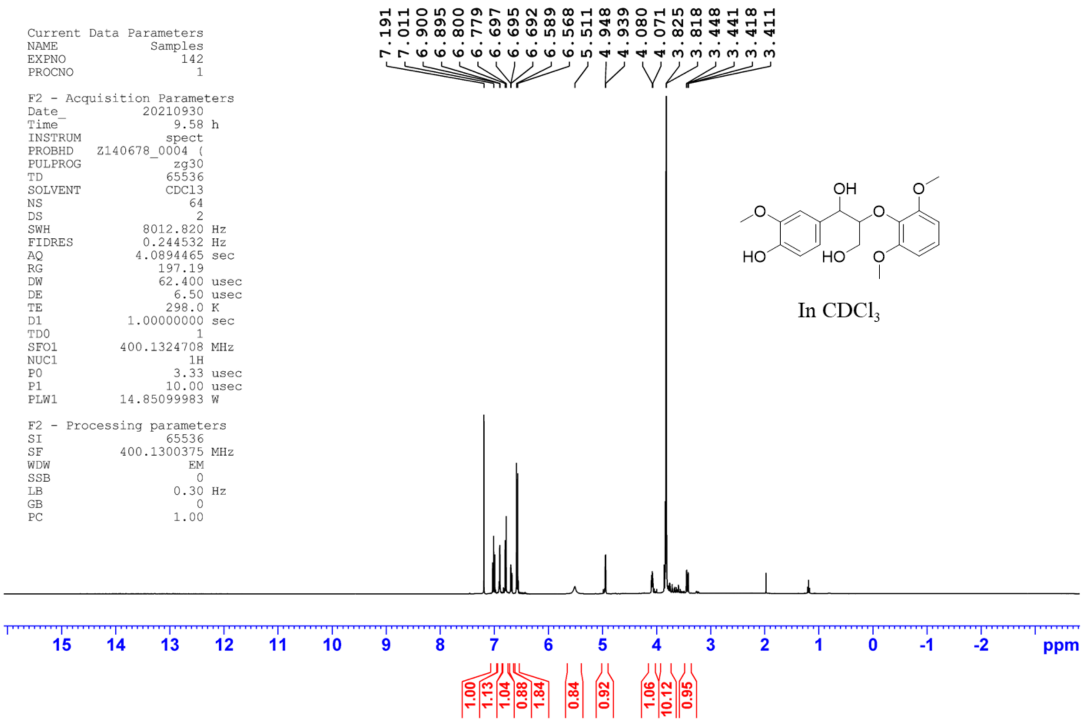

Supplementary Figure 25. ^{1}H NMR spectra of compound **k**.

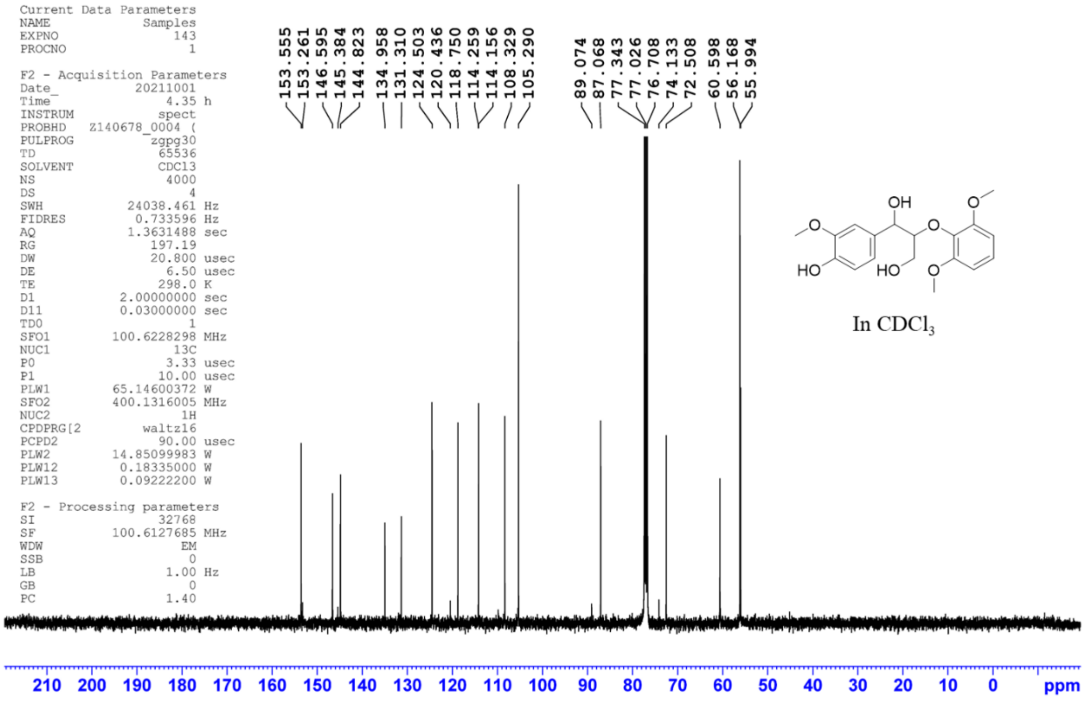

Supplementary Figure 26. ^{13}C NMR spectra of compound **k**.

NMR spectra of products:

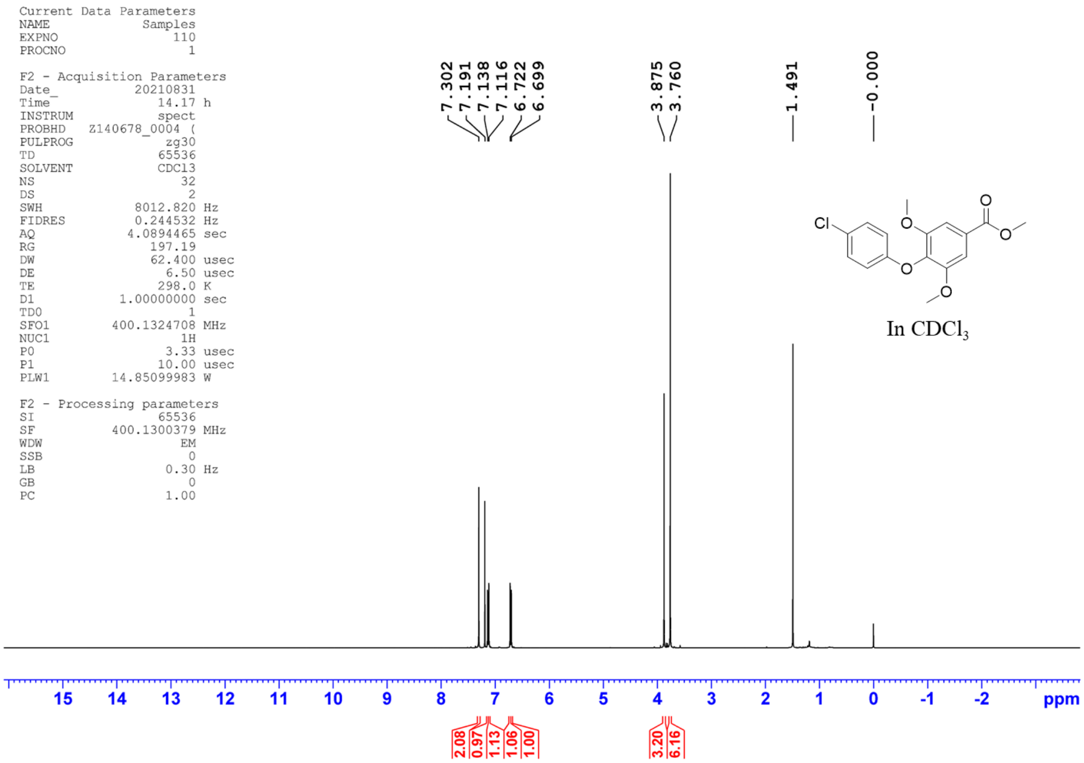

Supplementary Figure 27. ^{1}H NMR spectra of compound **1b**.

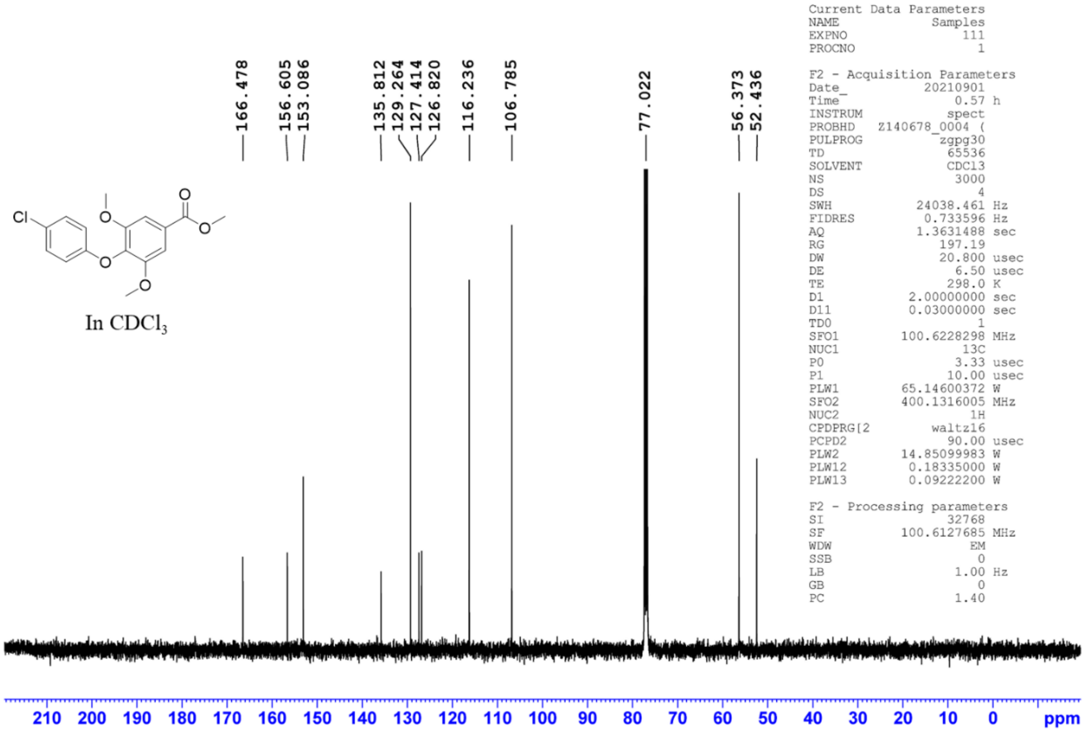

Supplementary Figure 28. ^{13}C NMR spectra of compound **1b**.

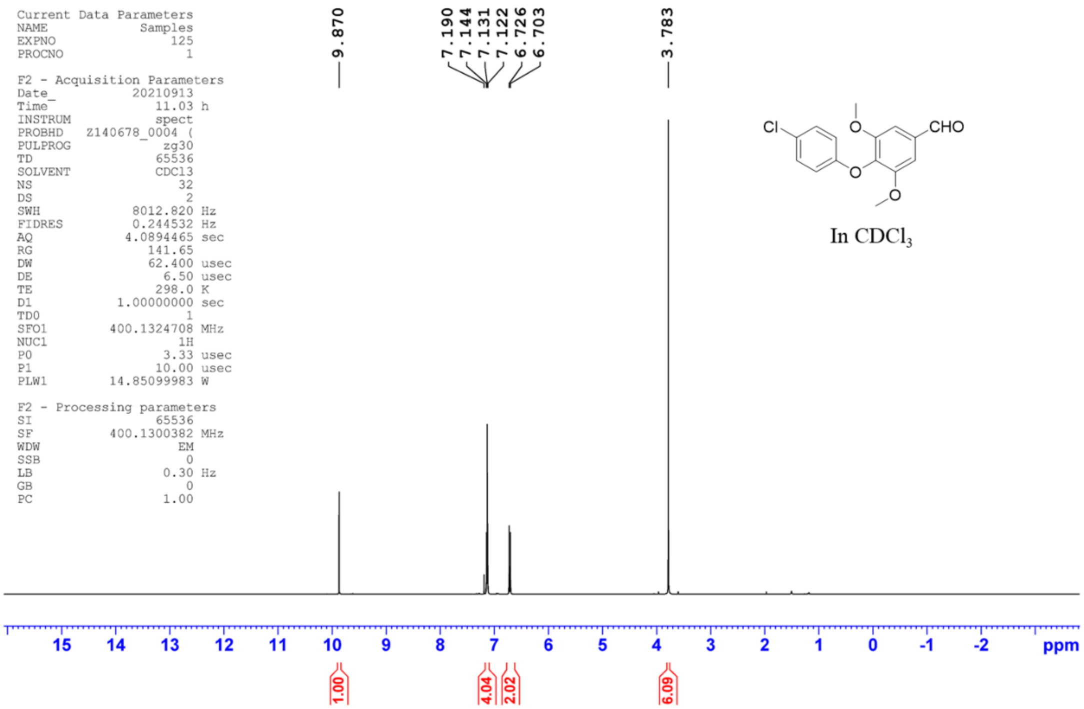

Supplementary Figure 29. ^{1}H NMR spectra of compound **2b**.

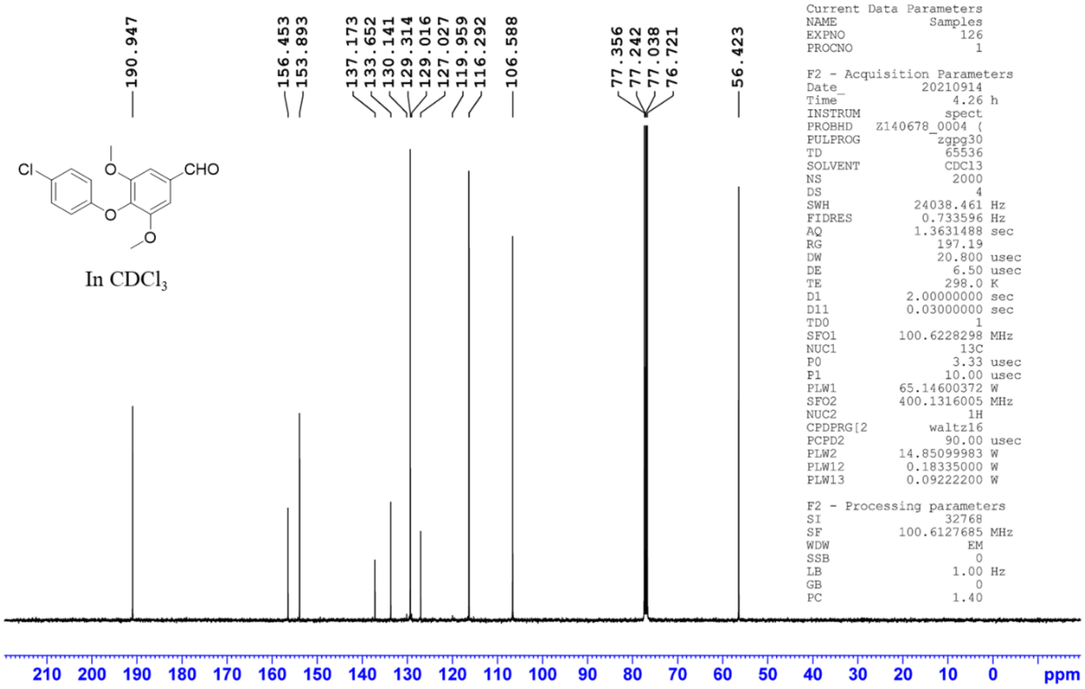

Supplementary Figure 30. ^{13}C NMR spectra of compound **2b**.

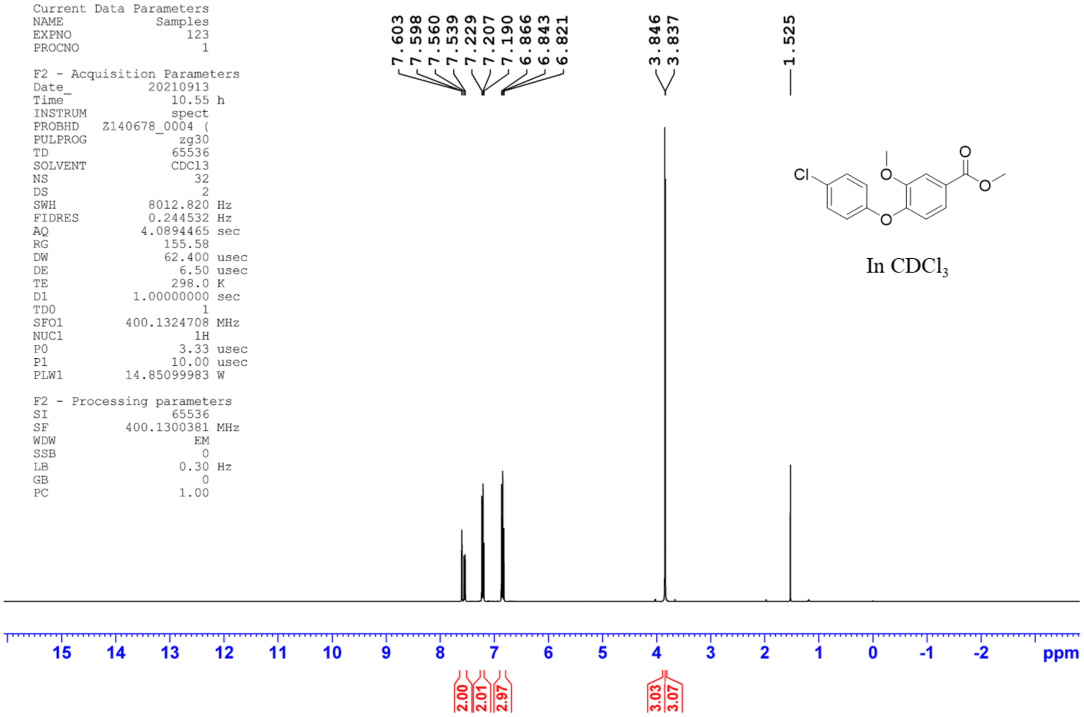

Supplementary Figure 31. ^{1}H NMR spectra of compound **3b**.

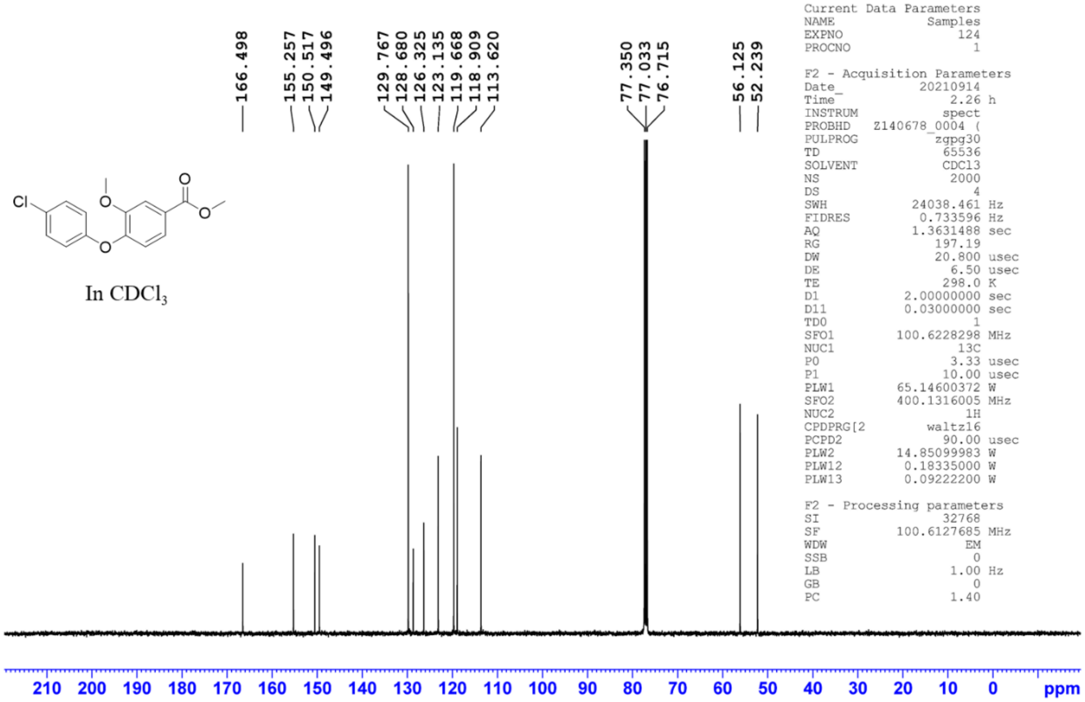

Supplementary Figure 32. ^{13}C NMR spectra of compound **3b**.

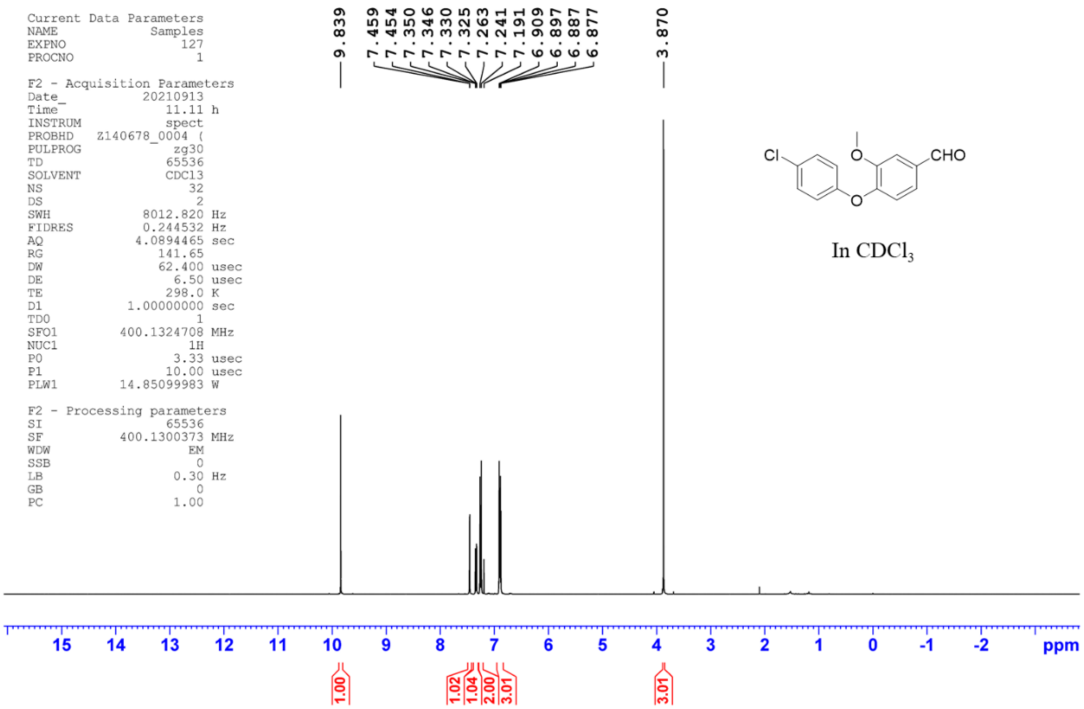

Supplementary Figure 33. ^{1}H NMR spectra of compound **4b**.

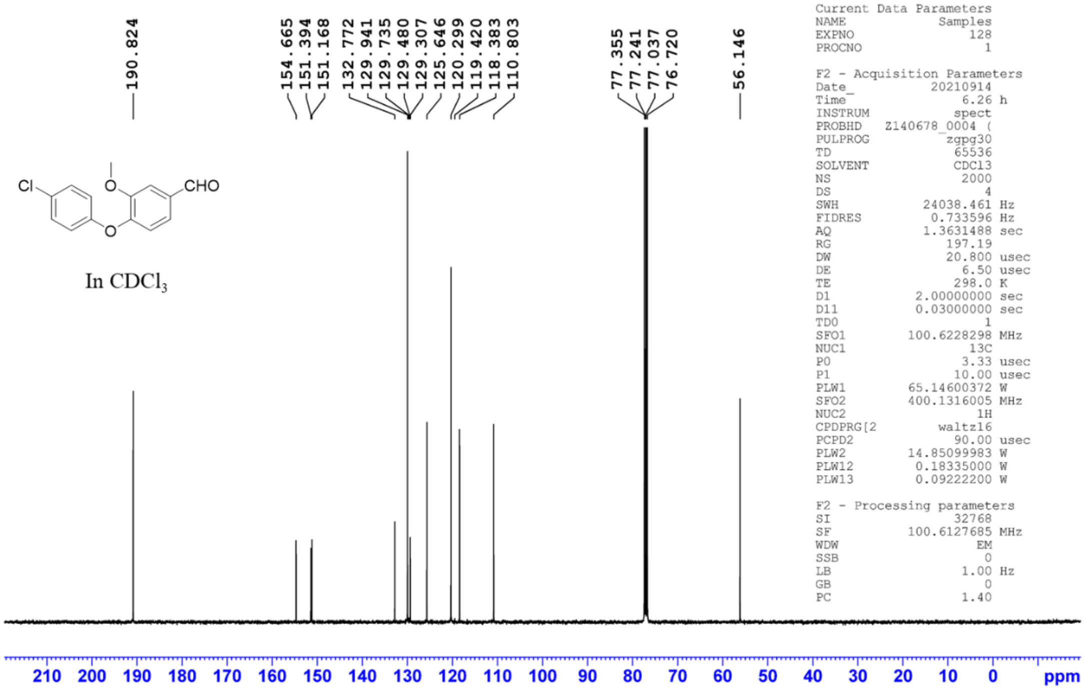

Supplementary Figure 34. ^{13}C NMR spectra of compound **4b**.

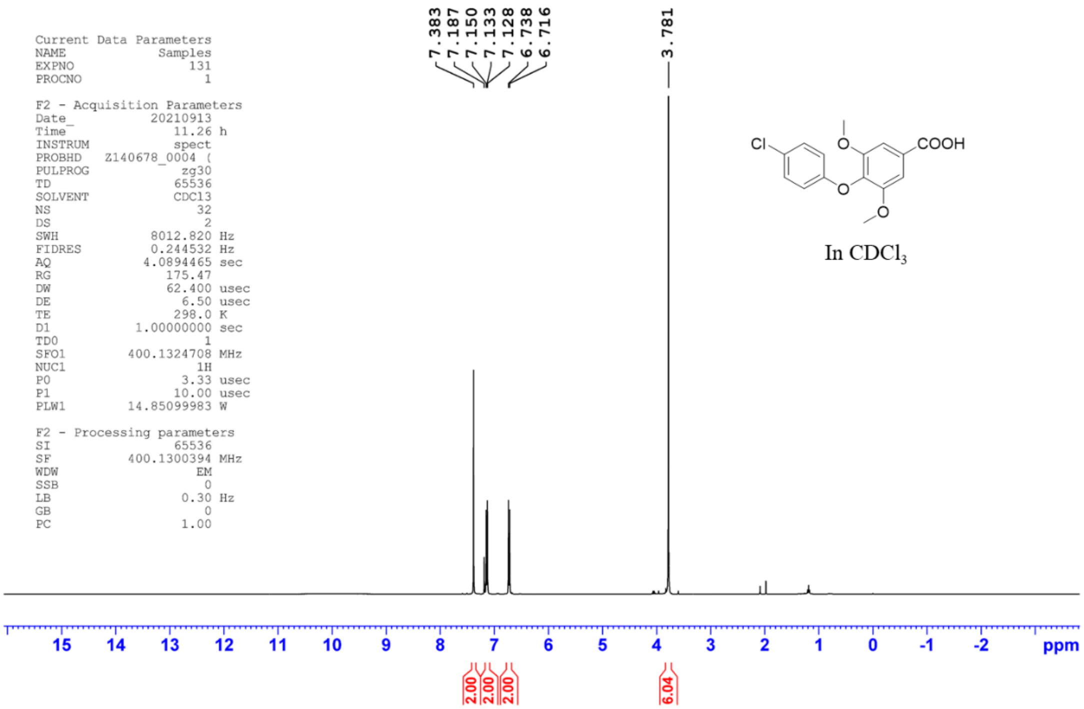

Supplementary Figure 35. ^{1}H NMR spectra of compound 4-(4-chlorophenoxy)-3,5-dimethoxybenzoic acid.

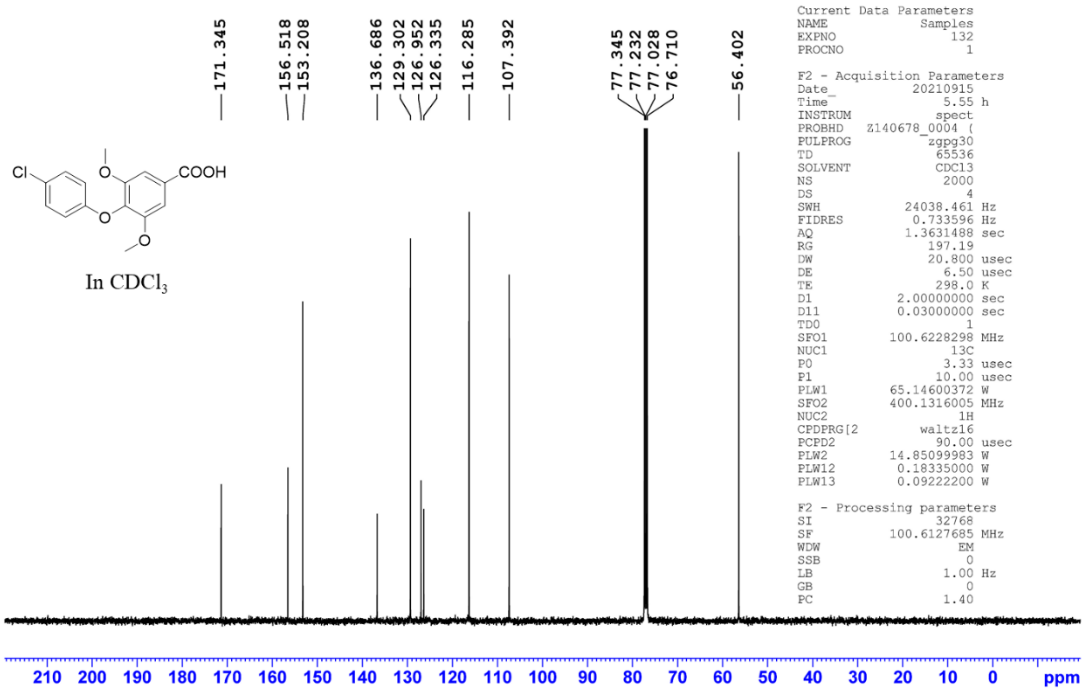

Supplementary Figure 36. ^{13}C NMR spectra of compound 4-(4-chlorophenoxy)-3,5-dimethoxybenzoic acid.

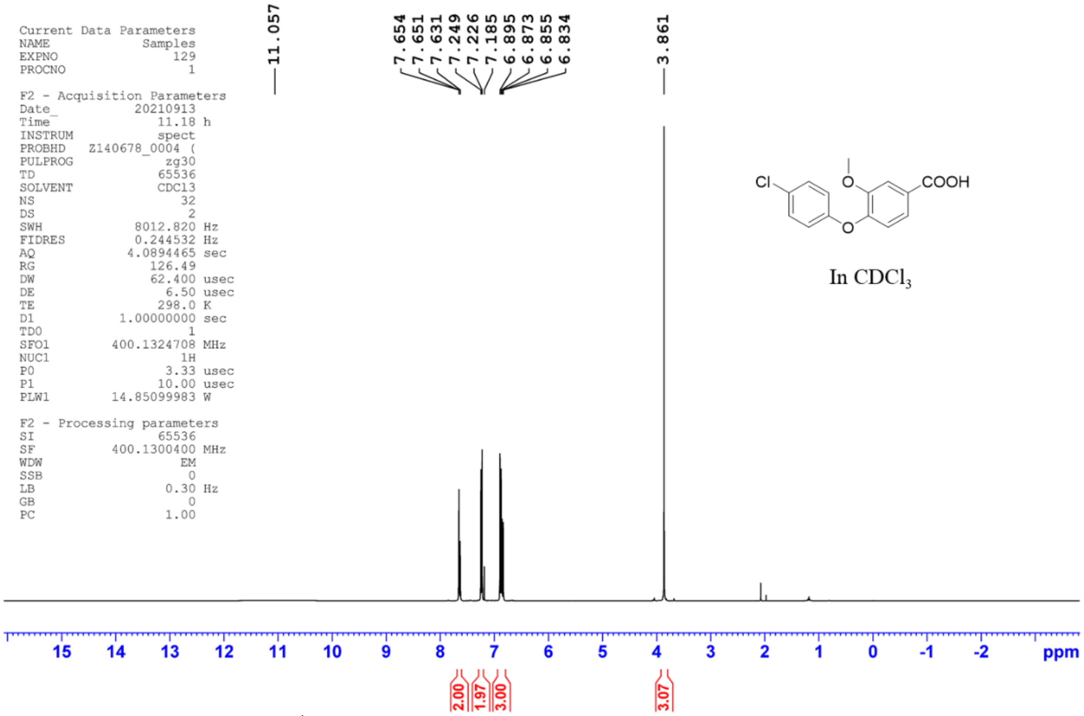

Supplementary Figure 37. ^{1}H NMR spectra of compound 4-(4-chlorophenoxy)-3-methoxybenzoic acid.

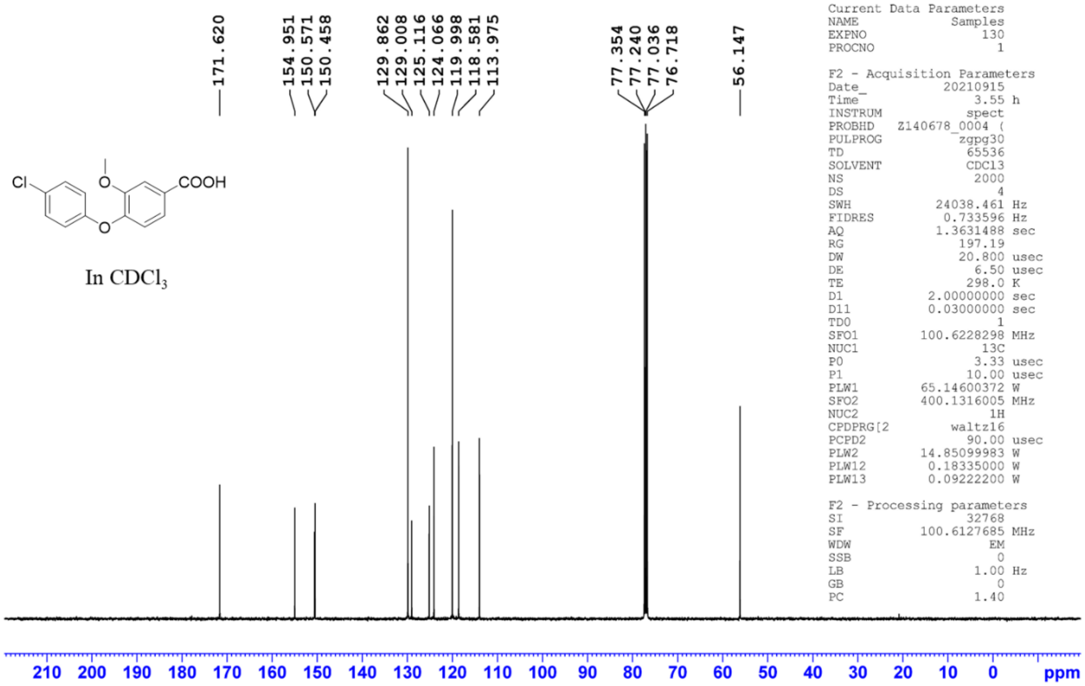

Supplementary Figure 38. ^{13}C NMR spectra of compound 4-(4-chlorophenoxy)-3-methoxybenzoic acid.

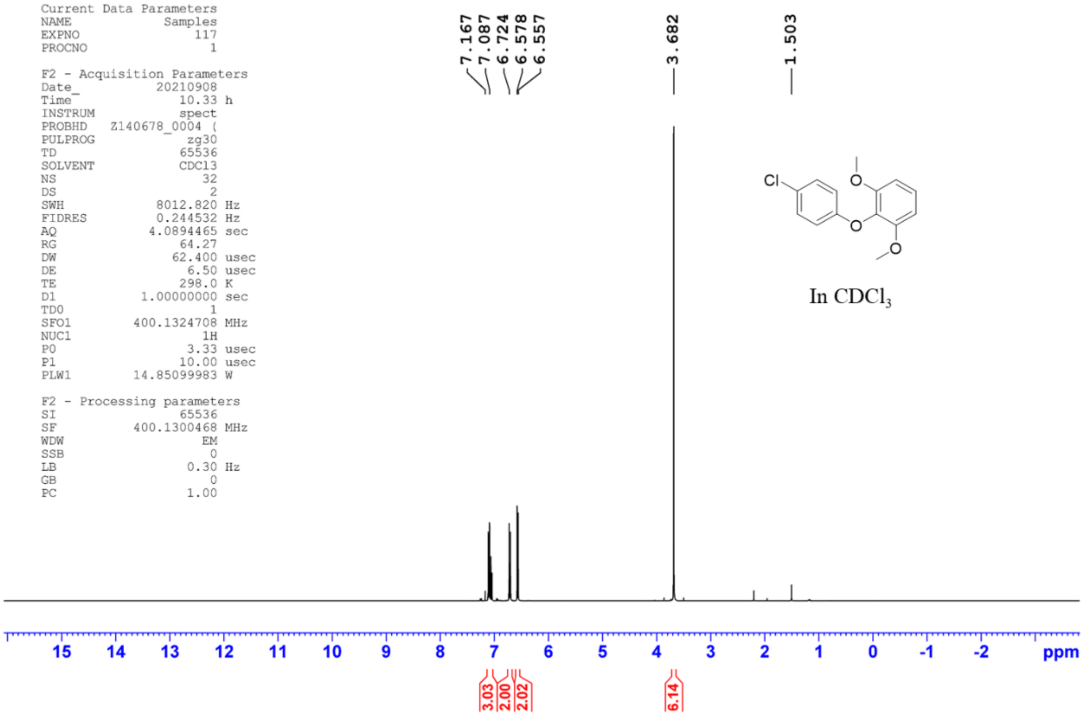

Supplementary Figure 39. ^{1}H NMR spectra of compound 2-(4-chlorophenoxy)-1,3-dimethoxybenzene.

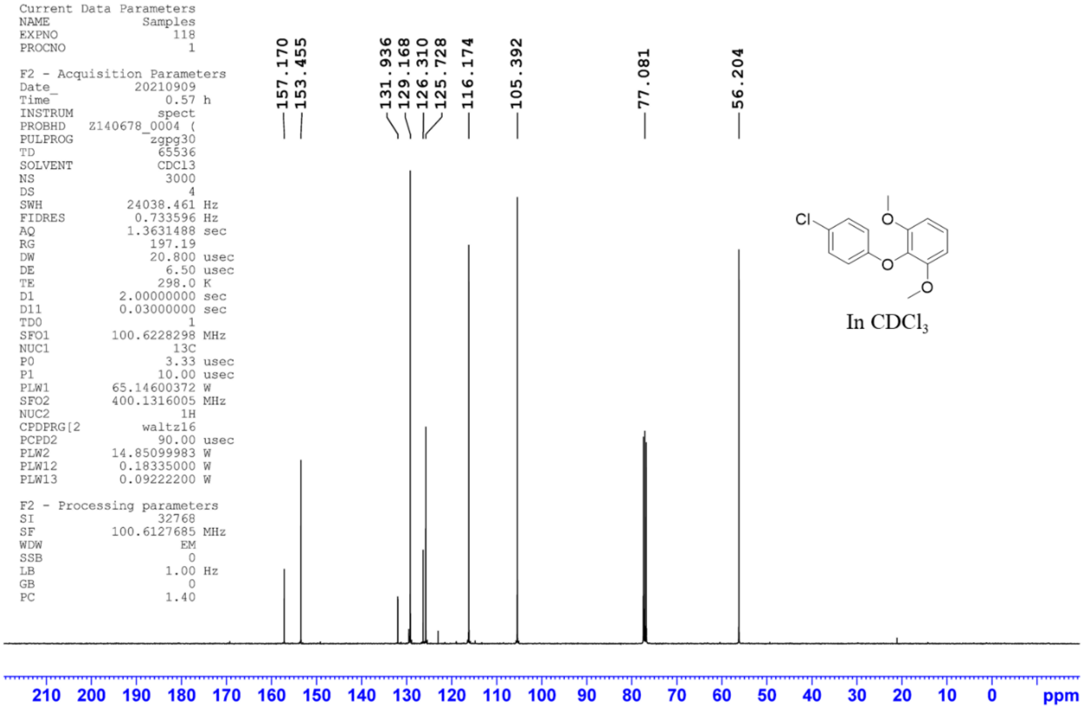

Supplementary Figure 40. ^{13}C NMR spectra of compound 2-(4-chlorophenoxy)-1,3-dimethoxybenzene.

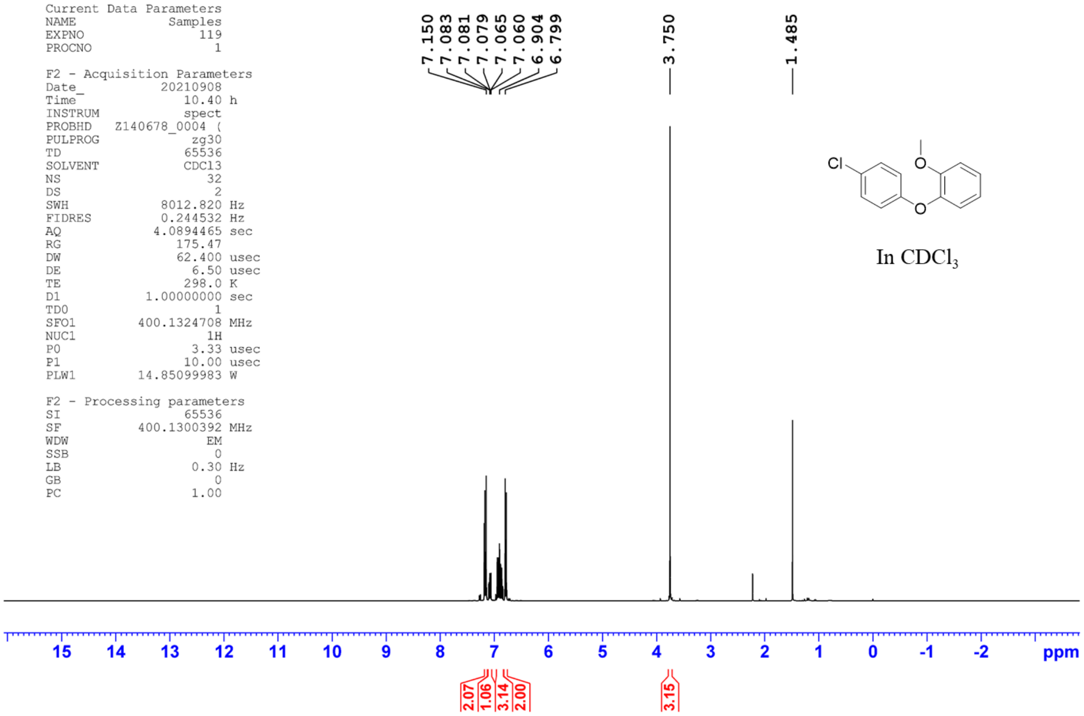

Supplementary Figure 41. ^{1}H NMR spectra of compound 2-Methoxy-4'-chlor-diphenylether.

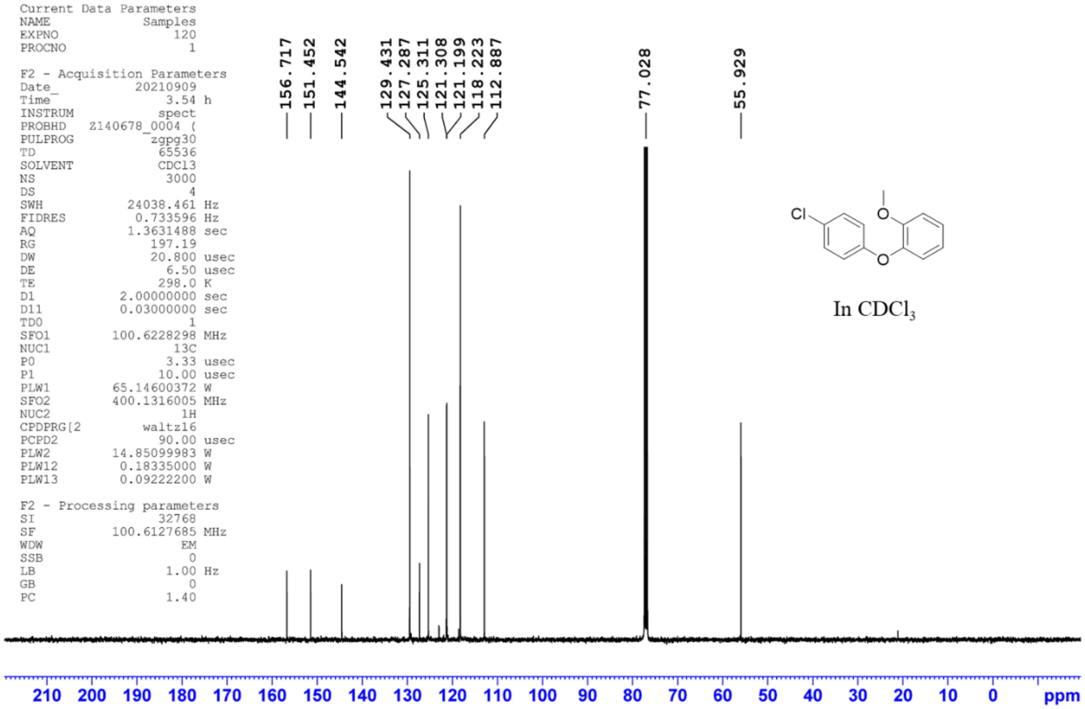

Supplementary Figure 42. ^{13}C NMR spectra of compound 2-Methoxy-4'-chlor-diphenylether.

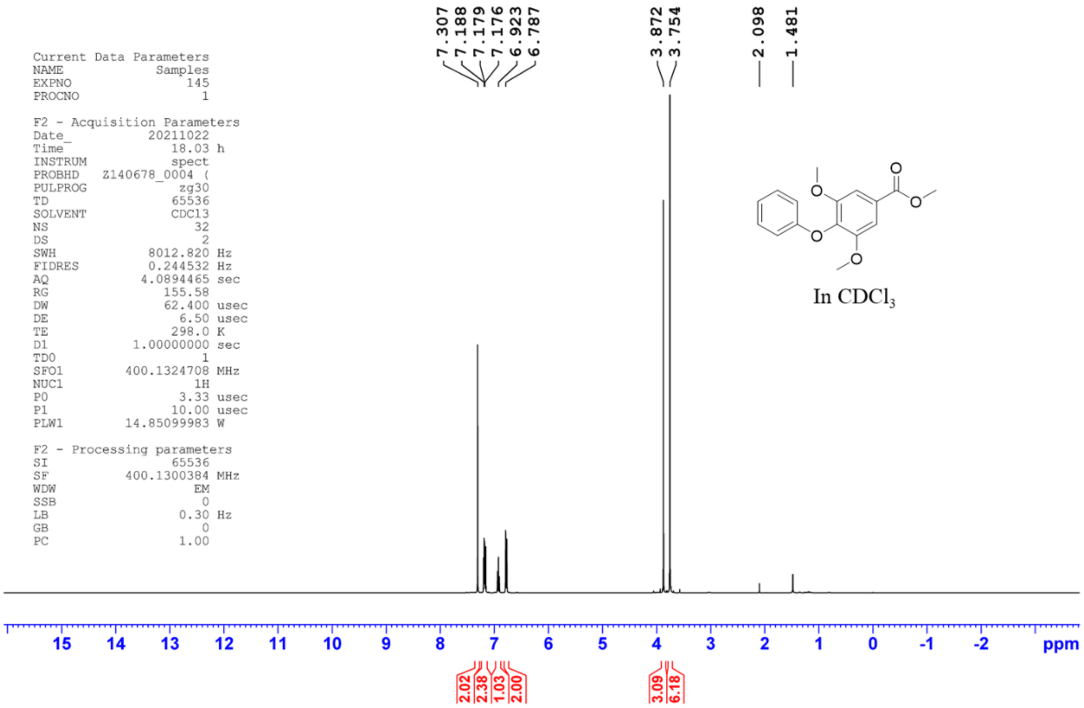

Supplementary Figure 43. ^{1}H NMR spectra of compound **6b**.

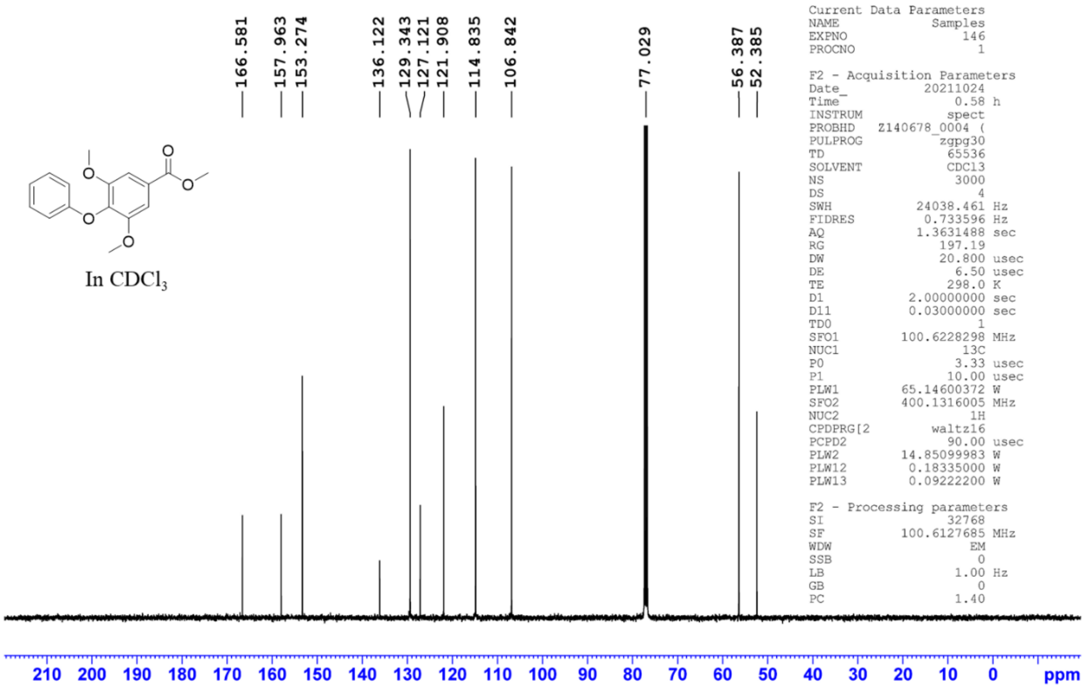

Supplementary Figure 44. ^{13}C NMR spectra of compound **6b**.

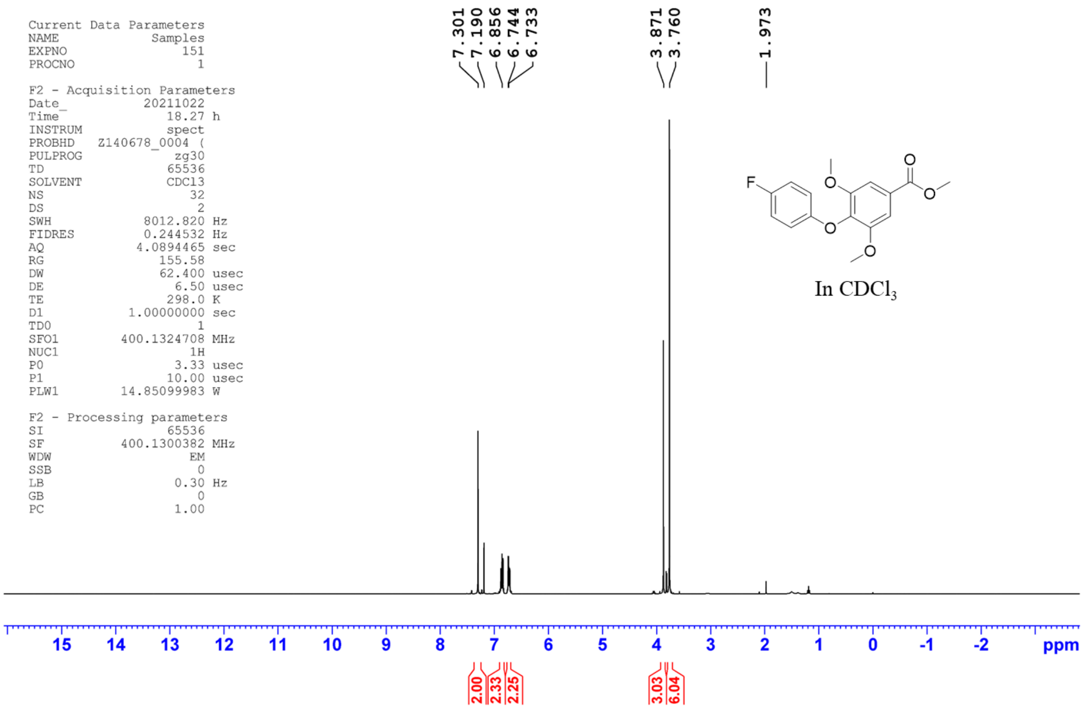

Supplementary Figure 45. ^{1}H NMR spectra of compound **7b**.

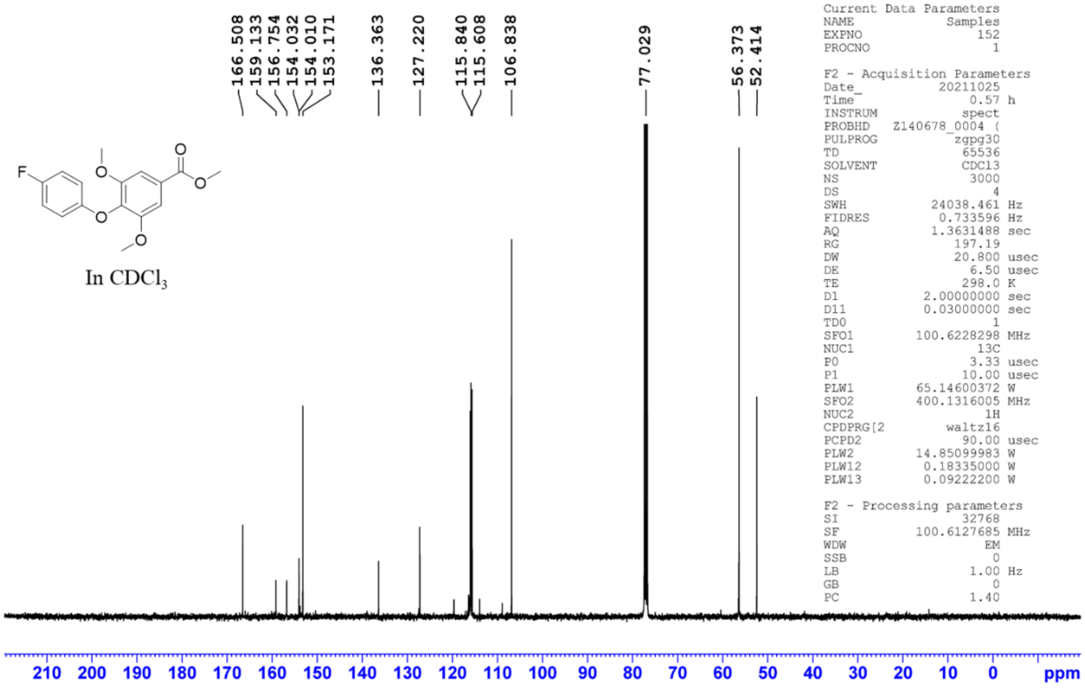

Supplementary Figure 46. ^{13}C NMR spectra of compound **7b**.

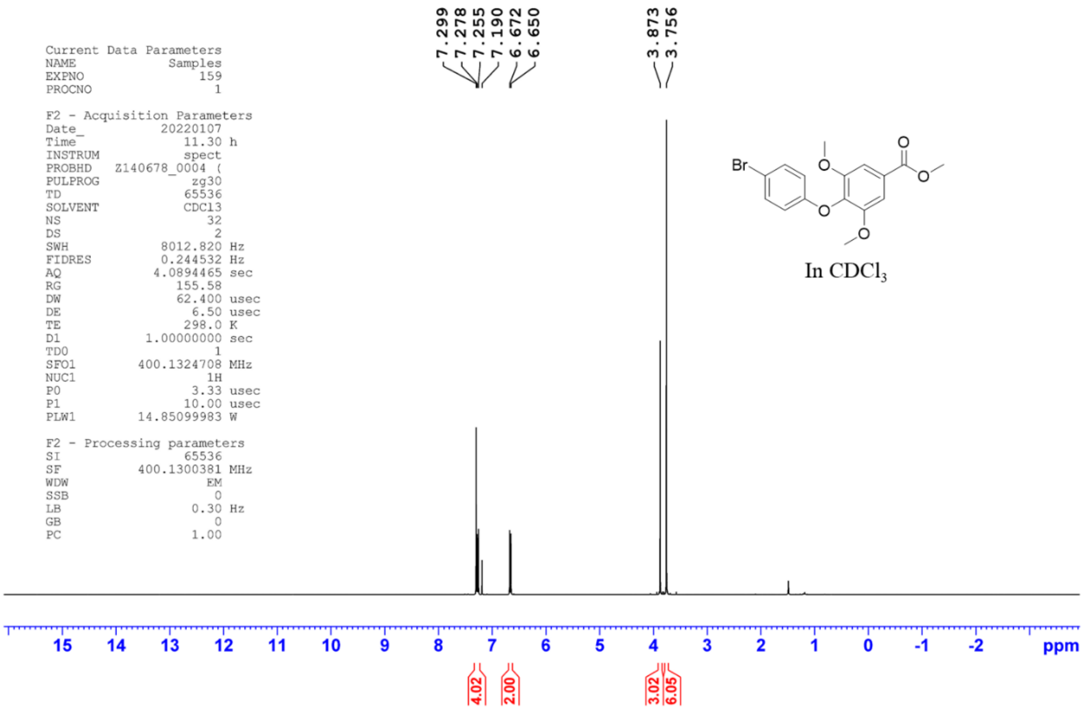

Supplementary Figure 47. ^{1}H NMR spectra of compound **8b**.

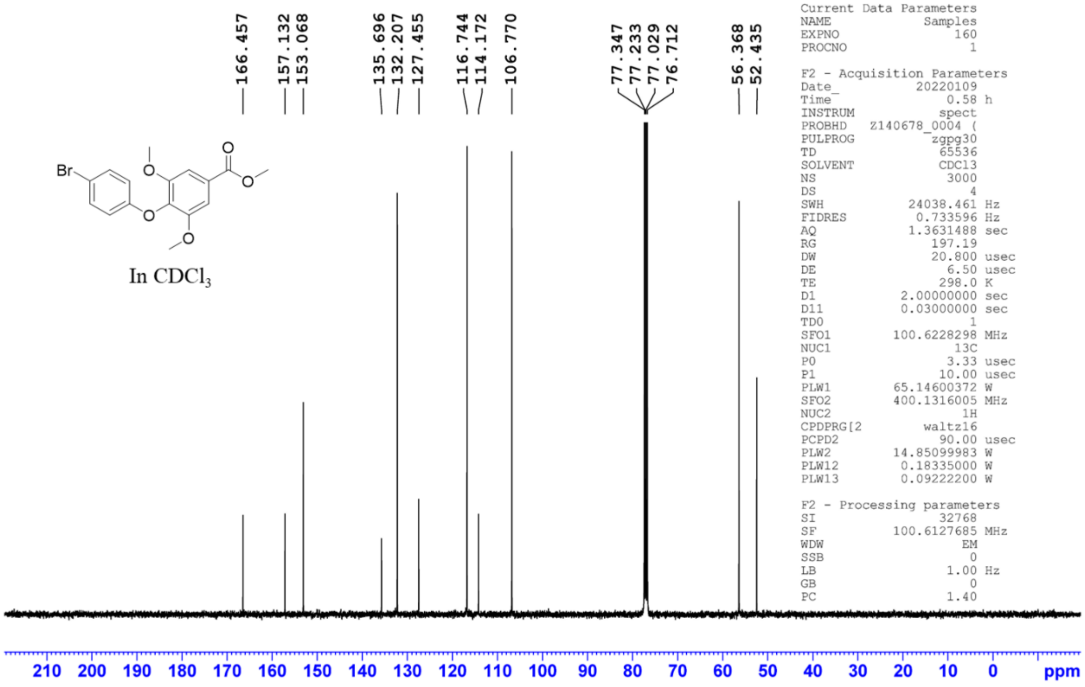

Supplementary Figure 48. ^{13}C NMR spectra of compound **8b**.

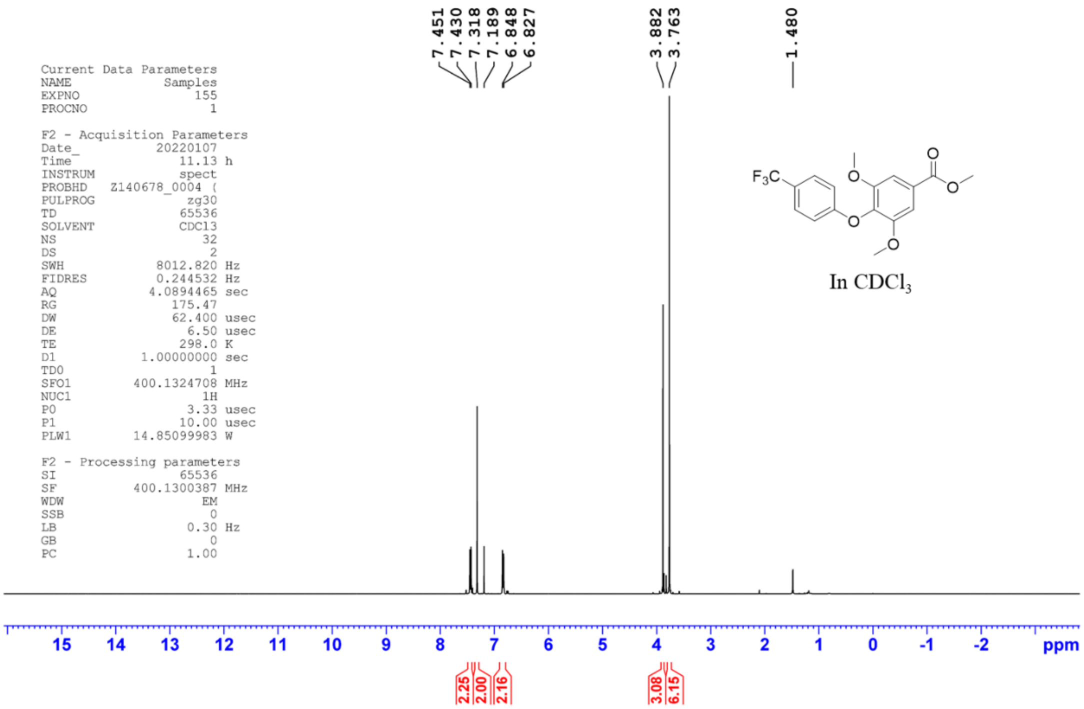

Supplementary Figure 49. ^{1}H NMR spectra of compound **9b**.

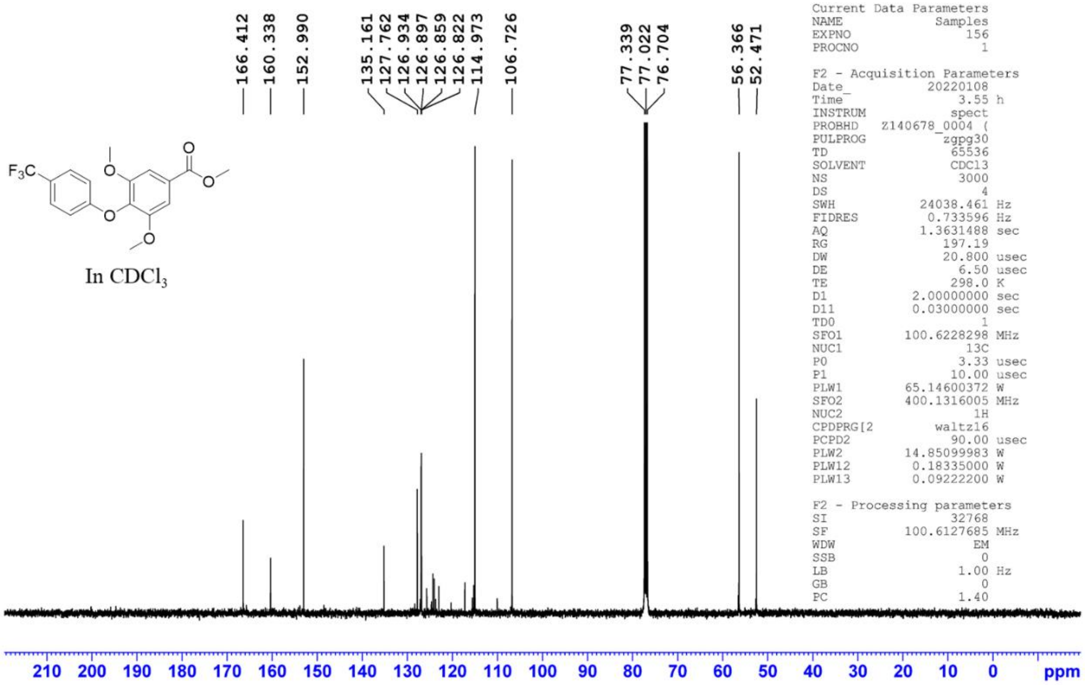

Supplementary Figure 50. ^{13}C NMR spectra of compound **9b**.

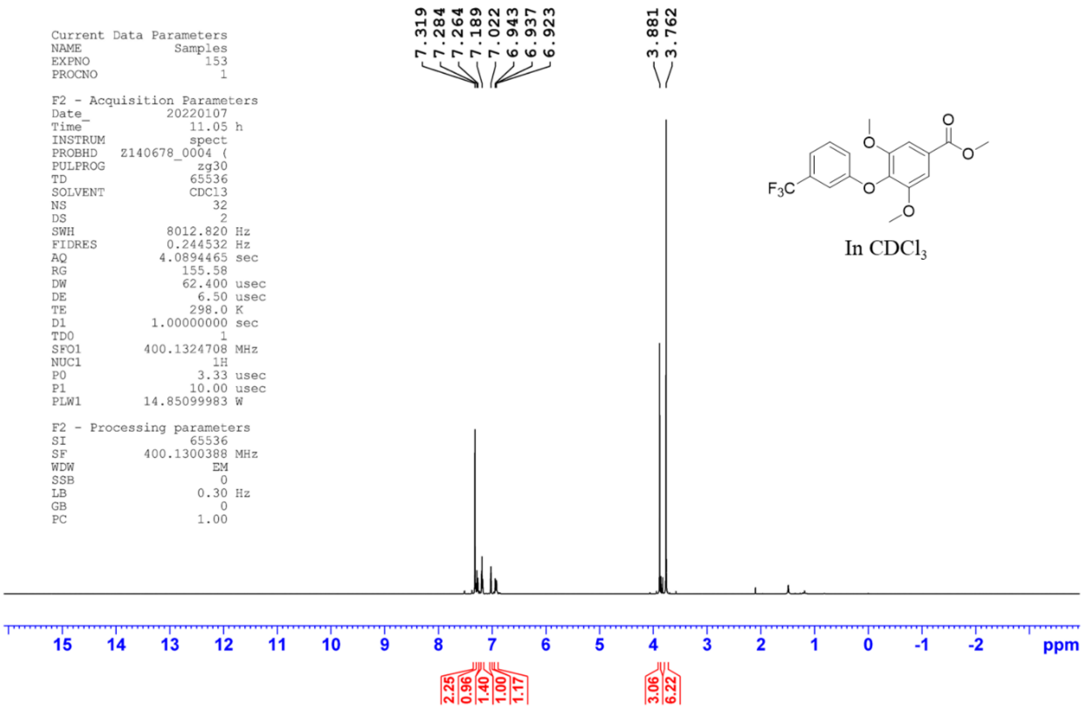

Supplementary Figure 51. ^{1}H NMR spectra of compound **10b**.

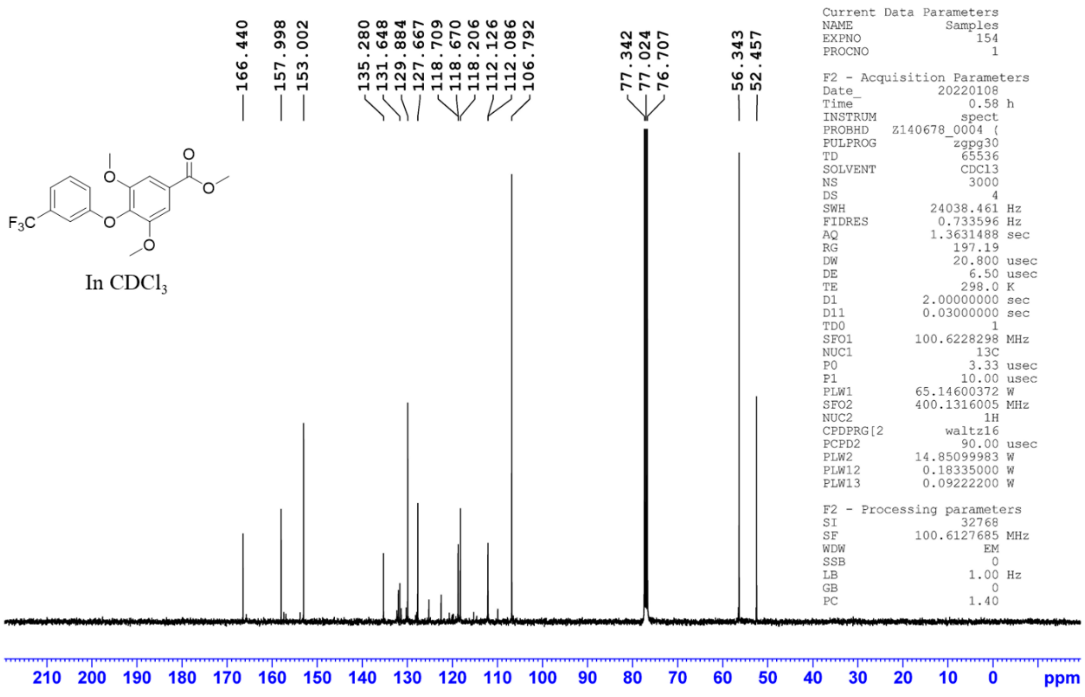

Supplementary Figure 52. ^{13}C NMR spectra of compound **10b**.

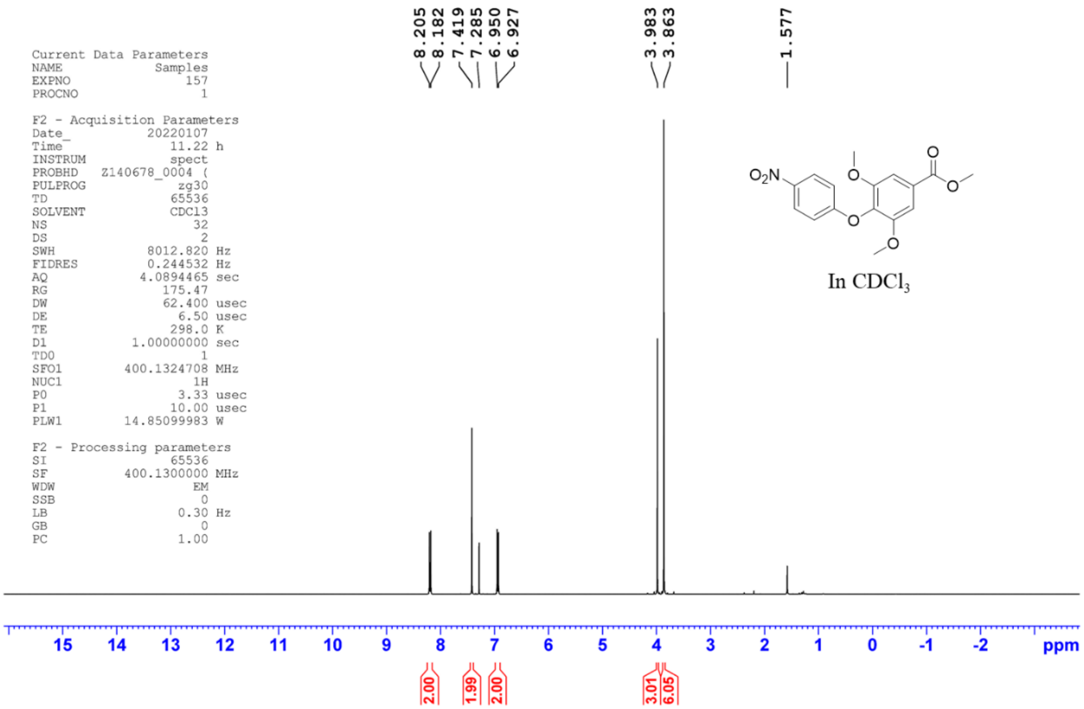

Supplementary Figure 53. ^{1}H NMR spectra of compound **11b**.

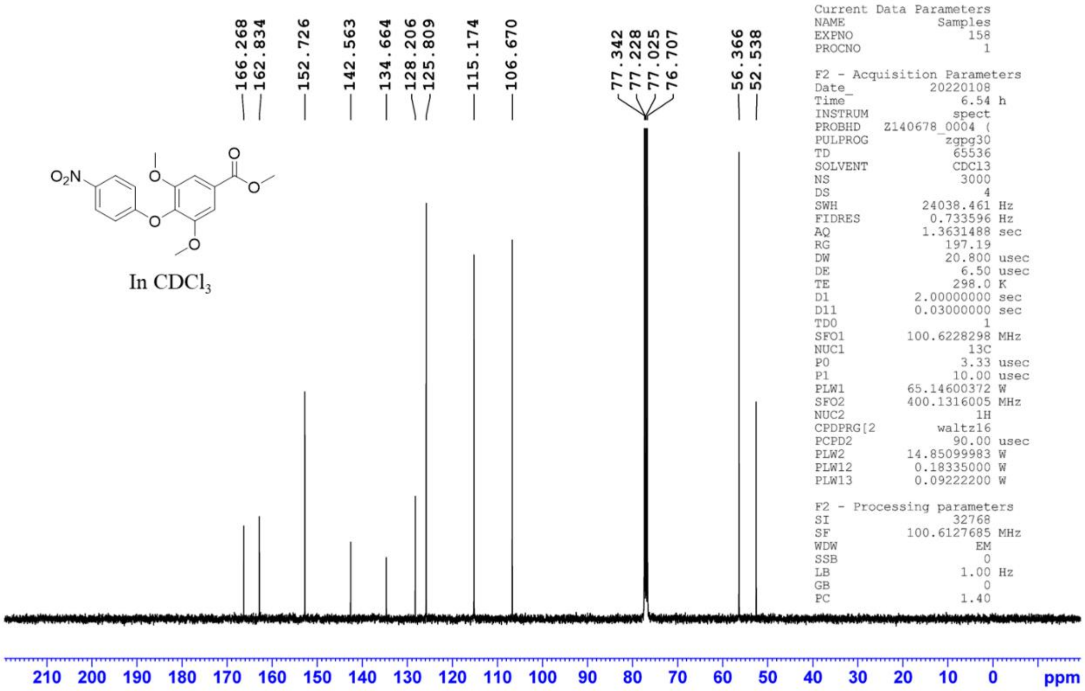

Supplementary Figure 54. ^{13}C NMR spectra of compound **11b**.

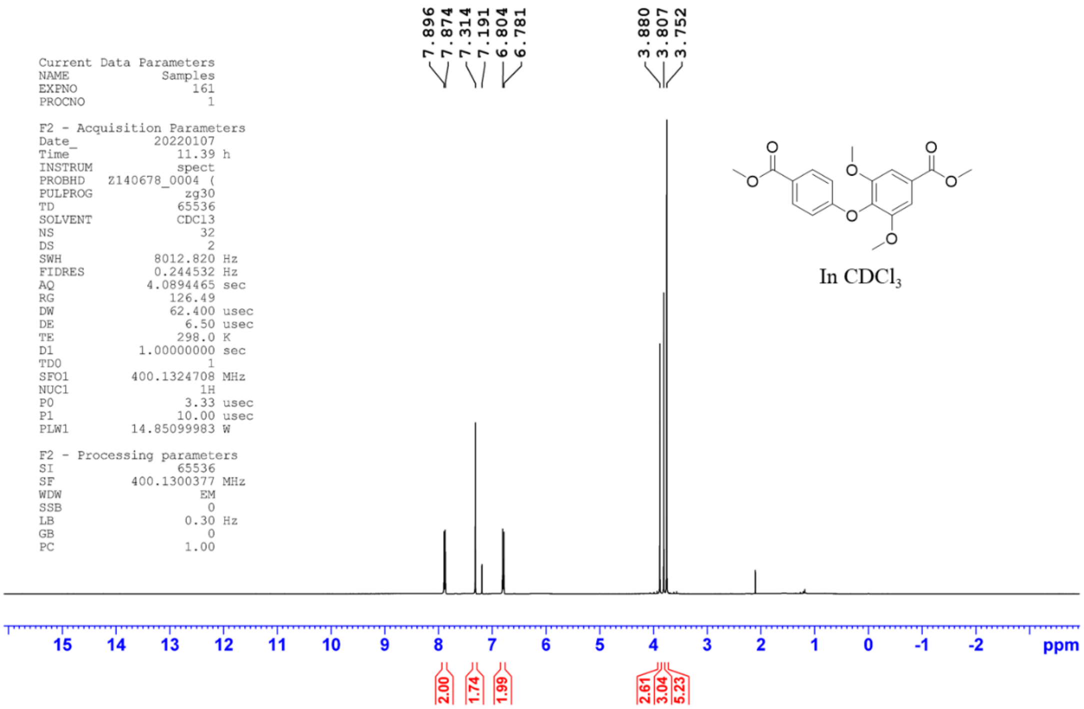

Supplementary Figure 55. ^{1}H NMR spectra of compound **12b**.

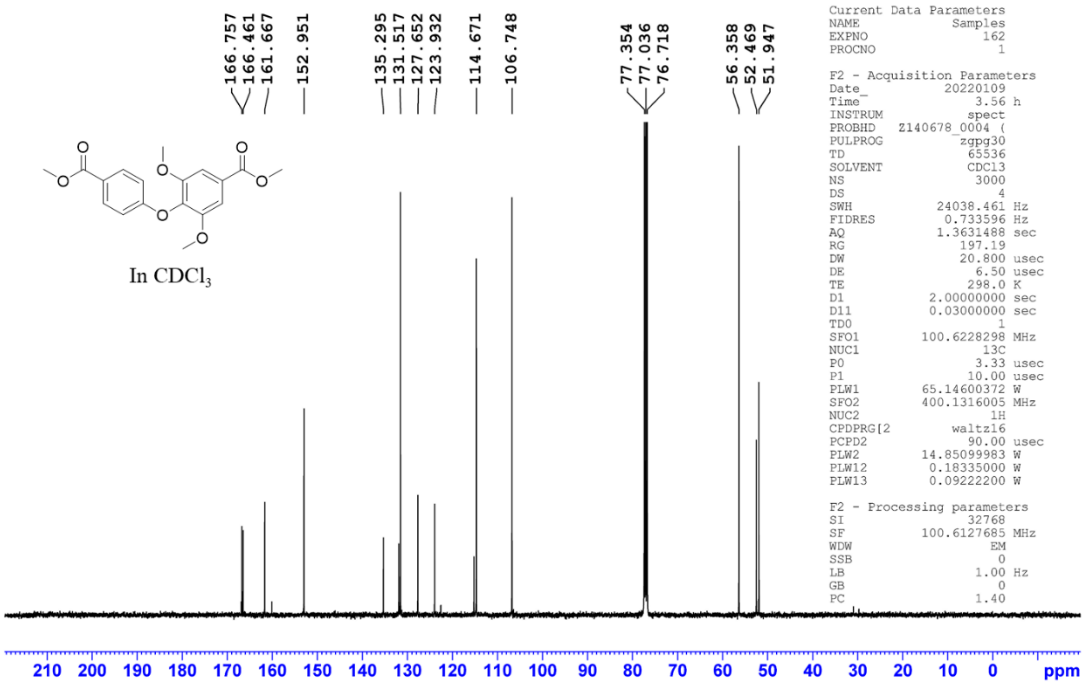

Supplementary Figure 56. ^{13}C NMR spectra of compound **12b**.

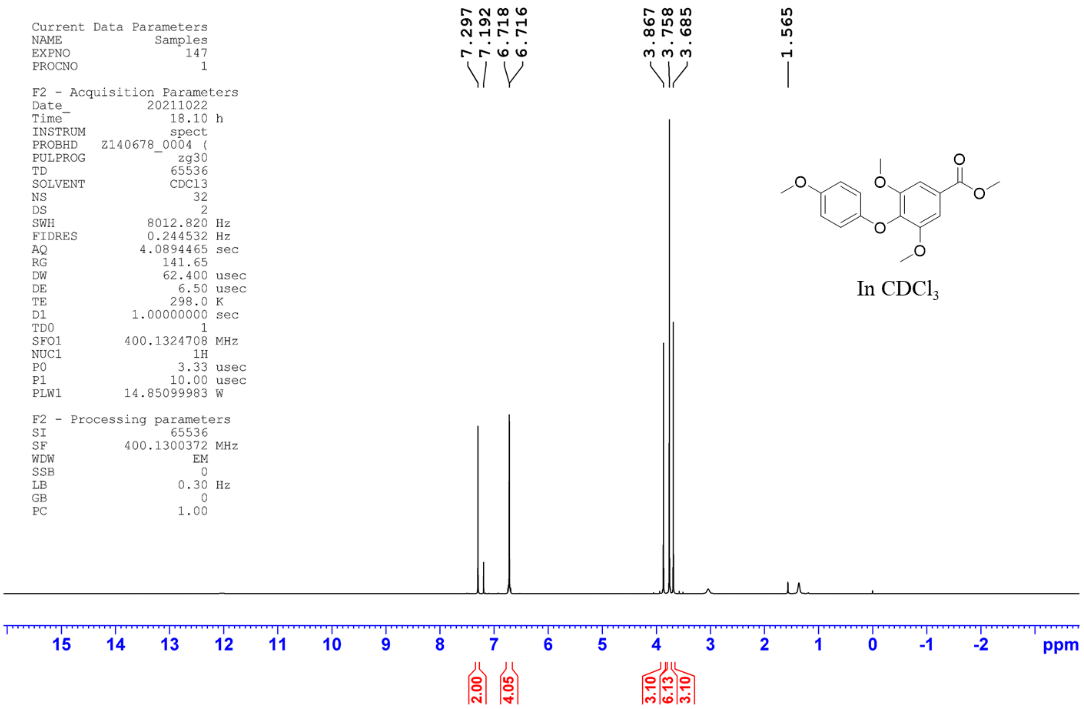

Supplementary Figure 57. ^{1}H NMR spectra of compound **13b**.

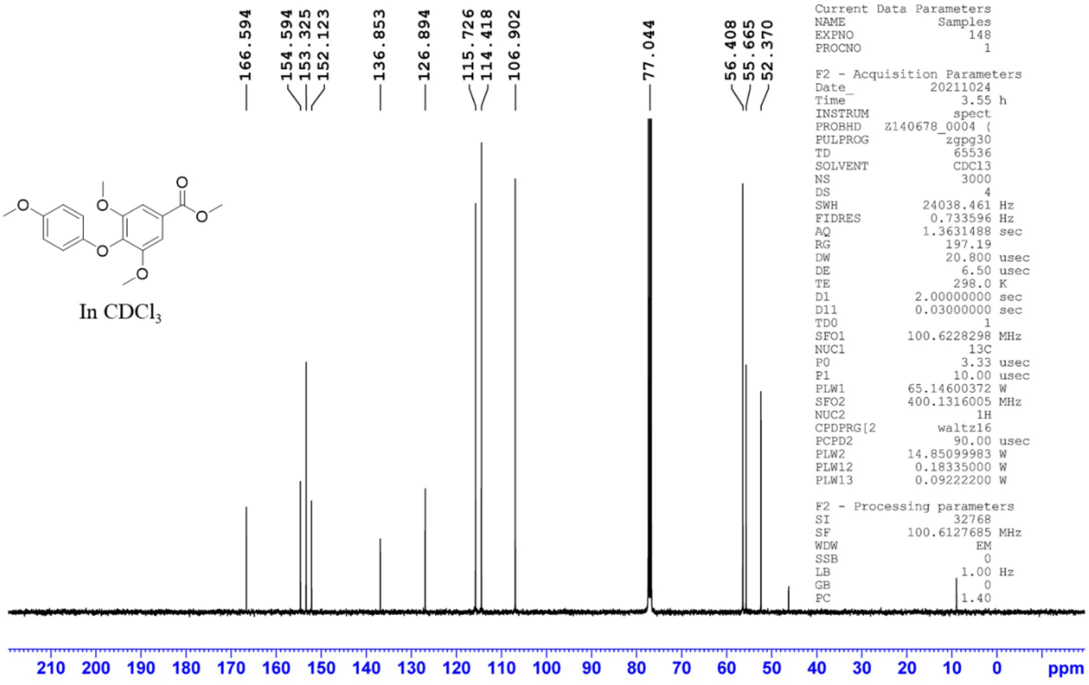

Supplementary Figure 58. ^{13}C NMR spectra of compound **13b**.

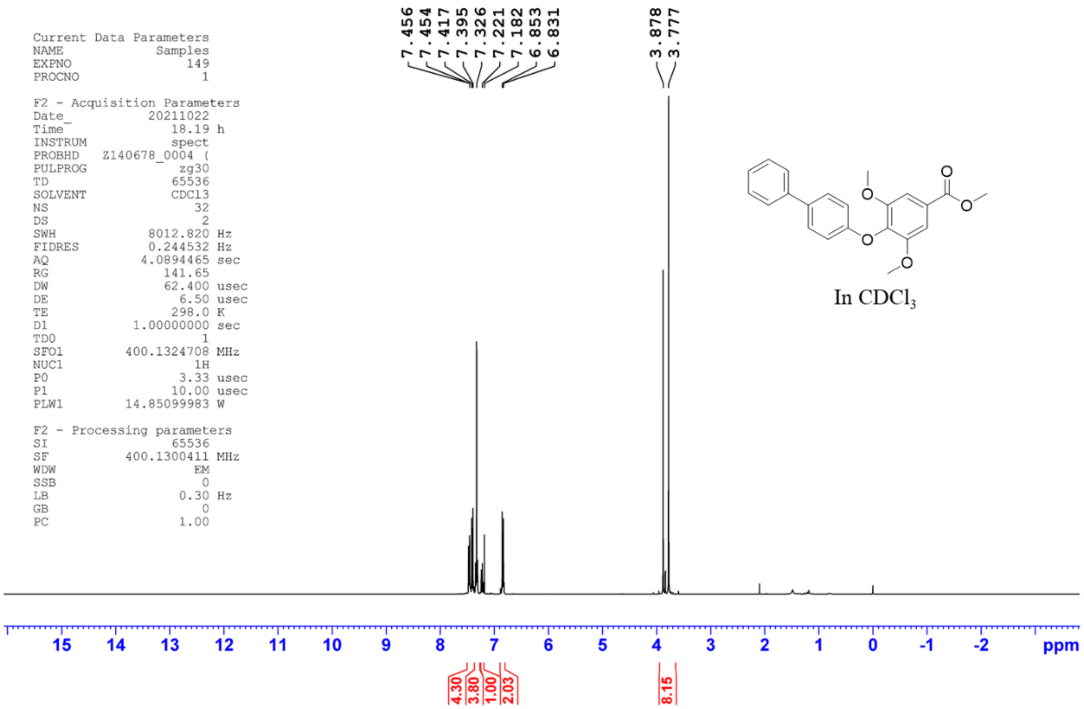

Supplementary Figure 59. ^{1}H NMR spectra of compound **14b**.

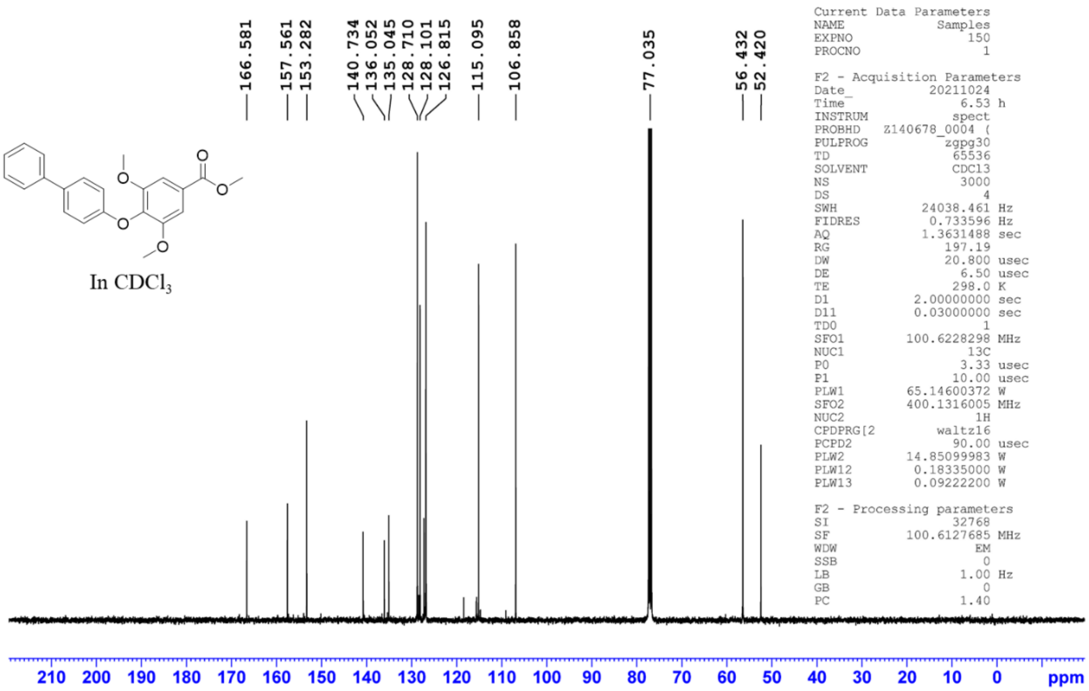

Supplementary Figure 60. ^{13}C NMR spectra of compound **14b**.

Supplementary References

1. Lancefield, C. S.; Ojo, O. S.; Tran, F.; Westwood, N. J., Isolation of Functionalized Phenolic Monomers through Selective Oxidation and C−O Bond Cleavage of the β-O-4 Linkages in Lignin. *Angew. Chem. Int. Ed.* **2015**, *54*, 258-262.
2. Lee, C. K.; Yu, J. S.; Lee, H.-J., Determination of aromaticity indices of thiophene and furan by nuclear magnetic resonance spectroscopic analysis of their phenyl esters. *J. Heterocycl. Chem.* **2002**, *39*, 1207-1217.
3. Ueda, T.; Konishi, H.; Manabe, K., Palladium-Catalyzed Carbonylation of Aryl, Alkenyl, and Allyl Halides with Phenyl Formate. *Org. Lett.* **2012**, *14*, 3100-3103.
4. Xia, Z.; Akim, L. G.; Argyropoulos, D. S., Quantitative ^{13}C NMR Analysis of Lignins with Internal Standards. *J. Agric. Food Chem.* **2001**, *49*, 3573-3578.
5. Yuan, T.-Q.; Sun, S.-N.; Xu, F.; Sun, R.-C., Characterization of Lignin Structures and Lignin–Carbohydrate Complex (LCC) Linkages by Quantitative ^{13}C and 2D HSQC NMR Spectroscopy. *J. Agric. Food Chem.* **2011**, *59*, 10604-10614.
6. Sette, M.; Lange, H.; Crestini, C., Quantitative HSQC analyses of lignin: a practical comparison. *Comput. Struct. Biotechnol. J.* **2013**, *6*, e201303016.
7. Azarpira, A.; Ralph, J.; Lu, F., Catalytic Alkaline Oxidation of Lignin and its Model Compounds: a Pathway to Aromatic Biochemicals. *Bioenergy Res.* **2014**, *7*, 78-86.
8. Ma, R.; Guo, M.; Lin, K.-t.; Hebert, V. R.; Zhang, J.; Wolcott, M. P.; Quintero, M.; Ramasamy, K. K.; Chen, X.; Zhang, X., Peracetic Acid Depolymerization of Biorefinery Lignin for Production of Selective Monomeric Phenolic Compounds. *Chem. Eur. J.* **2016**, *22*, 10884-10891.
9. Tarabanko, V. E.; Kaygorodov, K. L.; Skiba, E. A.; Tarabanko, N.; Chelbina, Y. V.; Baybakova, O. V.; Kuznetsov, B. N.; Djakovitch, L., Processing Pine Wood into Vanillin and Glucose by Sequential Catalytic Oxidation and Enzymatic Hydrolysis. *J. Wood Chem. Technol.* **2017**, *37*, 43-51.
10. Schutyser, W.; Kruger, J. S.; Robinson, A. M.; Katahira, R.; Brandner, D. G.; Cleveland, N. S.; Mittal, A.; Peterson, D. J.; Meilan, R.; Román-Leshkov, Y.; Beckham, G. T., Revisiting alkaline aerobic lignin oxidation. *Green Chem.* **2018**, *20*, 3828-3844.
11. Zhu, Y.; Liao, Y.; Lv, W.; Liu, J.; Song, X.; Chen, L.; Wang, C.; Sels, B. F.; Ma, L., Complementing Vanillin and Cellulose Production by Oxidation of Lignocellulose with Stirring Control. *ACS Sustain. Chem. Eng.* **2020**, *8*, 2361-2374.
12. Subbotina, E.; Rukkijakan, T.; Marquez-Medina, M. D.; Yu, X.; Johnsson, M.; Samec, J. S. M., Oxidative cleavage of C–C bonds in lignin. *Nat. Chem.* **2021**, *13*, 1118-1125.
13. Song, Y.; Mobley, J. K.; Motagamwala, A. H.; Isaacs, M.; Dumesic, J. A.; Ralph, J.; Lee, A. F.; Wilson, K.; Crocker, M., Gold-catalyzed conversion of lignin to low molecular weight aromatics. *Chem. Sci.* **2018**, *9*, 8127-8133.
14. Cai, Z.; Long, J.; Li, Y.; Ye, L.; Yin, B.; France, L. J.; Dong, J.; Zheng, L.; He, H.; Liu, S.; Tsang, S. C. E.; Li, X., Selective Production of Diethyl Maleate via Oxidative Cleavage of Lignin Aromatic Unit. *Chem* **2019**, *5*, 2365-2377.
15. Voß, D.; Pickel, H.; Albert, J., Improving the Fractionated Catalytic Oxidation of Lignocellulosic Biomass to Formic Acid and Cellulose by Using Design of Experiments. *ACS Sustain. Chem. Eng.* **2019**, *7*, 9754-9762.
16. Luo, H.; Weeda, E. P.; Alherech, M.; Anson, C. W.; Karlen, S. D.; Cui, Y.; Foster, C. E.; Stahl, S. S., Oxidative Catalytic Fractionation of Lignocellulosic Biomass under Non-alkaline Conditions. *J. Am. Chem. Soc.* **2021**, *143*, 15462-15470.
17. Du, X.; Tricker, A. W.; Yang, W.; Katahira, R.; Liu, W.; Kwok, T. T.; Gogoi, P.; Deng, Y., Oxidative Catalytic Fractionation and Depolymerization of Lignin in a One-Pot Single-Catalyst System. *ACS Sustain. Chem. Eng.* **2021**, *9*, 7719-7727.
18. Ryland, B. L.; Stahl, S. S., Practical Aerobic Oxidations of Alcohols and Amines with Homogeneous Copper/TEMPO and Related Catalyst Systems. *Angew. Chem. Int. Ed.* **2014**, *53*, 8824-8838.

19. Liu, M.; Li, C.-J., Catalytic Fehling's Reaction: An Efficient Aerobic Oxidation of Aldehyde Catalyzed by Copper in Water. *Angew. Chem. Int. Ed.* **2016**, *55*, 10806-10810.
20. Wang, M.; Lu, J.; Li, L.; Li, H.; Liu, H.; Wang, F., Oxidative C(OH)−C bond cleavage of secondary alcohols to acids over a copper catalyst with molecular oxygen as the oxidant. *J. Catal.* **2017**, *348*, 160-167.
21. Huang, X.; Li, X.; Zou, M.; Song, S.; Tang, C.; Yuan, Y.; Jiao, N., From Ketones to Esters by a Cu-Catalyzed Highly Selective C(CO)−C(alkyl) Bond Cleavage: Aerobic Oxidation and Oxygenation with Air. *J. Am. Chem. Soc.* **2014**, *136*, 14858-14865.
22. Liang, Y.-F.; Jiao, N., Oxygenation via C−H/C−C Bond Activation with Molecular Oxygen. *Acc. Chem. Res.* **2017**, *50*, 1640-1653.
23. Chang, J.-G.; Chen, H.-T.; Xu, S.; Lin, M. C., Computational Study on the Kinetics and Mechanisms for the Unimolecular Decomposition of Formic and Oxalic Acids. *J. Phys. Chem. A* **2007**, *111*, 6789-6797.
24. Molt, R. W.; Lecher, A. M.; Clark, T.; Bartlett, R. J.; Richards, N. G. J., Facile C_{sp2}–C_{sp2} Bond Cleavage in Oxalic Acid-Derived Radicals. *J. Am. Chem. Soc.* **2015**, *137*, 3248-3252.
